# Improving the communication of multifactorial cancer risk assessment results for different audiences: a co-design process

**DOI:** 10.1007/s12687-024-00729-4

**Published:** 2024-09-25

**Authors:** Francisca Stutzin Donoso, Tim Carver, Lorenzo Ficorella, Nichola Fennell, Antonis C. Antoniou, Douglas F. Easton, Marc Tischkowitz, Fiona M. Walter, Juliet A. Usher-Smith, Stephanie Archer

**Affiliations:** 1 Department of Public Health and Primary Care, University of Cambridge, Cambridge, UK; 2 Department of Medical Genetics, National Institute for Health Research Cambridge Biomedical Research Centre, University of Cambridge, Cambridge, UK; 3 Wolfson Institute of Population Health, Barts and the London School of Medicine and Dentistry, Queen Mary University of London, London, UK; 4 Department of Psychology, University of Cambridge, Cambridge, UK

**Keywords:** Breast cancer, Multifactorial cancer risk assessment, Co-design, Risk communication

## Abstract

**Background:**

Multifactorial cancer risk prediction tools, such as CanRisk, are increasingly being incorporated into routine healthcare. Understanding risk information and communicating risk is challenging and healthcare professionals rely substantially on the outputs of risk prediction tools to communicate results. This work aimed to produce a new CanRisk report so users can directly access key information and communicate risk estimates effectively.

**Methods:**

Over a 13-month period, we led an 8-step co-design process with patients, the public, and healthcare professionals. Steps comprised 1) think aloud testing of the original CanRisk report; 2) structured feedback on the original report; 3) literature review; 4) development of a new report prototype; 5) first round of structured feedback; 6) updating the new report prototype; 7) second round of structured feedback; and 8) finalising and publishing the new CanRisk report.

**Results:**

We received 56 sets of feedback from 34 stakeholders. Overall, the original CanRisk report was not suitable for patients and the public. Building on the feedback, the new report has an overview of the information presented: section one summarises key information for individuals; sections two and three present information for healthcare professionals in different settings. New features also include explanatory text, definitions, graphs, keys and tables to support the interpretation of the information.

**Discussion:**

This co-design experience shows the value of collaboration for the successful communication of complex health information. As a result, the new CanRisk report has the potential to better support shared decision-making processes about cancer risk management across clinical settings.

**Supplementary Information:**

The online version contains supplementary material available at 10.1007/s12687-024-00729-4.

## Introduction

Multifactorial cancer risk prediction tools such as CanRisk (www.canrisk.org), are increasingly being incorporated into routine healthcare. The CanRisk tool (CanRisk.org) uses the BOADICEA model (Lee et al. 2019, 2022), which is endorsed by the National Institute for Health and Care Excellence (NICE) (National Institute for Health and Care Excellence 2017) and other clinical management guidelines. The BOADICEA model combines individual data on family history, demographic, lifestyle, and hormonal risk factors, rare pathogenic genetic variants in cancer susceptibility genes, common genetic susceptibility variants (in the form of polygenic scores) and mammographic density, to calculate the probability of someone developing breast cancer or ovarian cancer in the future (Lee et al. 2019, 2022; Pal Choudhury et al. 2021; Yang et al. 2022). Since the CanRisk tool received its CE mark in early 2020 (Carver et al. 2021), over 2 million breast and ovarian cancer risk calculations have been completed worldwide. Even though the tool is available for use by healthcare professionals free of charge across clinical settings, it is predominantly used in clinical genetics and secondary care.

Recent studies have focused on testing the usability and acceptability of CanRisk among clinicians from a variety of settings (Archer et al. 2020), and on identifying the barriers and facilitators of its use in primary care (Archer & Stutzin Donoso et al. 2023). One of the key barriers to its implementation in primary care is the lack of confidence and knowledge about cancer risk prediction among healthcare professionals in this context (Archer & Stutzin Donoso et al. 2023). Primary care healthcare professionals in particular rely substantially on how results are presented in risk outputs or reports to communicate risk to people undergoing assessments (Vassy et al. 2018). More generally, research shows that the presentation of risk information (i.e., format and content of risk output or report) impacts on implementation and shared decision-making, as it affects how clinicians use the tool and how people undergoing the assessment understand and perceive their risk (Brigden et al. 2023). Thus, ensuring that the CanRisk report offers adequate support for individuals and healthcare professionals across clinical settings to interpret and communicate risk outcomes is paramount for realising the benefits of the tool in practice.

Understanding and communicating risk outcomes can be difficult. Besides adequately understanding risk information, healthcare professionals face the challenge of contextualising the relevant information and communicating it effectively considering individual circumstances and needs (Julian-Reynier et al. 2003). Risk perceptions can vary significantly depending on the communication strategies used, and these may not always support decision-making or health desired behavioural change (Goldman et al. 2006; Woof et al. 2023). Despite there not being one ‘right way’ of communicating risk, there is substantial research that has generated recommendations about how to best communicate health risk across groups such as using visual support, avoiding verbal communications only (Brigden et al. 2023; Richter et al. 2023), using icon arrays, absolute risk measures, risk-adjusted age graphs and personalised communications (Farmer et al. 2020; Glanz et al. 2015; Goldman et al. 2006; Lewis et al. 2022; Richter et al. 2023).

**Fig. 1:**
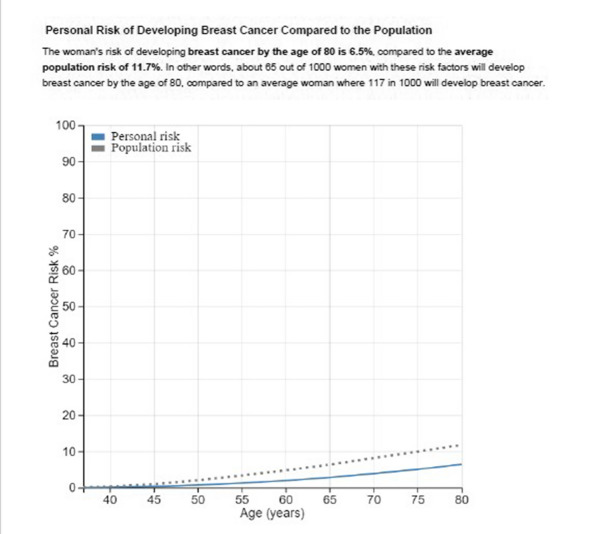
Personal risk of developing breast cancer compared to the population

**Fig. 2:**
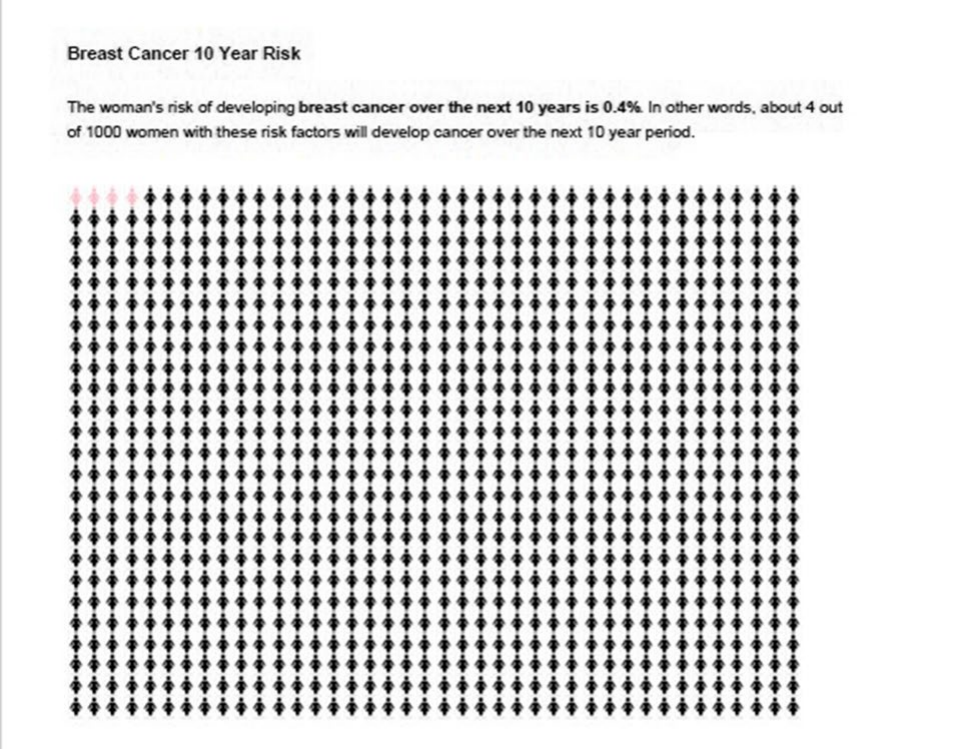
Breast cancer 10 year risk

The original version of the CanRisk report provided healthcare professionals with information to support communication but was not designed to fulfil the requirements of a self-explanatory report that could be shared with individuals undergoing the assessment. Examples from the visual representations provided by the original report are presented in Figs. 1 and 2.

As the CanRisk tool becomes used more widely, there is an emerging need to re-design the CanRisk report so it i) makes key information about the risk of developing breast and ovarian cancer more accessible to individuals undergoing the assessment, and ii) adequately supports healthcare professionals across clinical settings to use the tool and communicate results clearly and accurately. Here we describe a co-design process that aims to produce a new CanRisk report that addresses these challenges.

## Methods

### Design

**Fig. 3:**
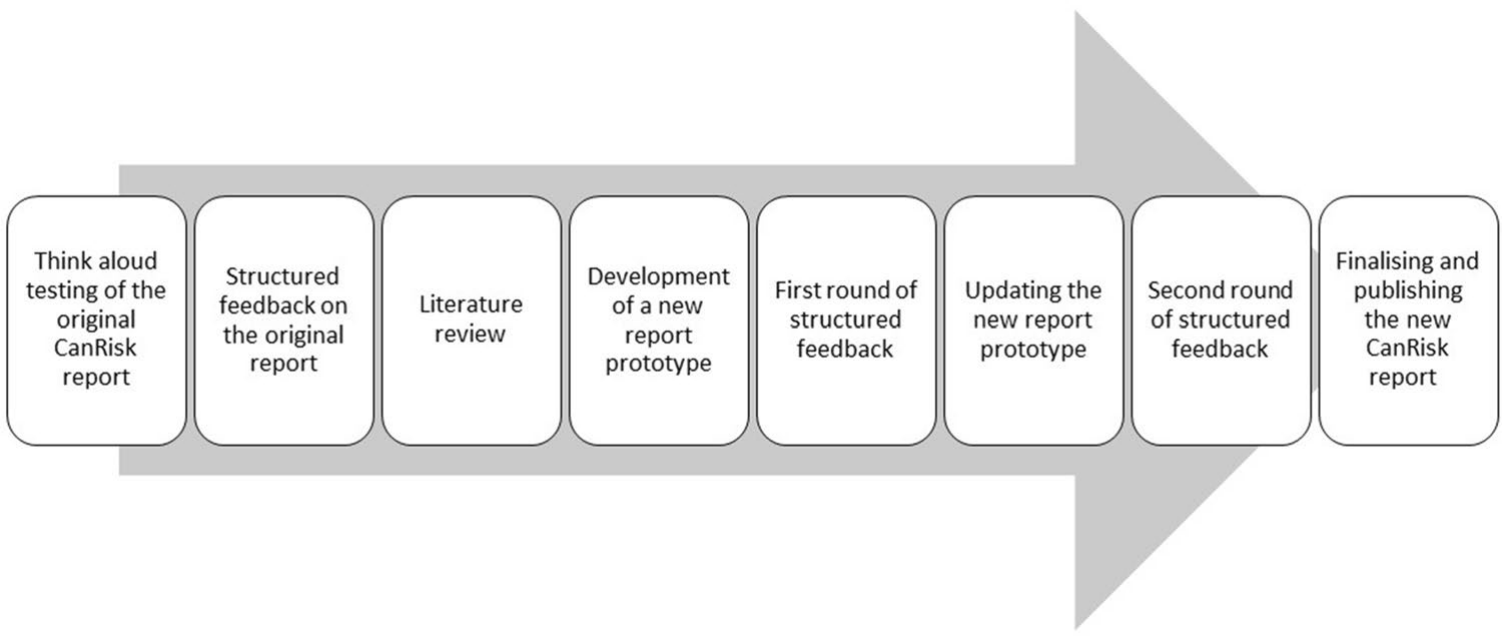
Overview of our co-design process

Between September 2022 and October 2023, we led an 8-step co-design (Realpe & Wallace 2010) process with stakeholders including patients, members of the public, and healthcare professionals with training in primary care and/or clinical genetics (see Fig. 3). ‘Co-design’ draws on the concept of co-production in healthcare and refers to the active collaboration between stakeholders in the design of solutions to a defined problem (Realpe and Wallace 2010). This method has been argued to increase the diversity of ideas, the quality of the product and the users’ experience (Vargas et al. 2022), and can reduce risks of error and development/maintenance costs (Vargas et al. 2022). Ethical approval for a wider project was granted by the Psychology Ethics Committee at the University of Cambridge, REF: 3531.132.

#### Think-aloud testing of the original CanRisk report

A pre-existing group of six patients and members of the public took part in a think-aloud (Eccles and Arsal 2017) testing session of the original CanRisk report in September 2022. The original group were recruited via social media and research networks to inform the design of a public facing version of the CanRisk tool. The group were purposively selected to achieve a heterogenous sample covering different age groups, gender, level of education and employment status (details shown in supplementary Table 1). After generating a CanRisk report using data from a fictitious individual for a breast cancer risk assessment (Appendix 1), we asked participants to read through the report out loud and share their thoughts on each aspect of the report with a researcher. After completing this task, all participants were prompted to share their main likes and dislikes about the report in a researcher-led facilitated discussion. Content analysis (Elo and Kyngäs, 2008) was used to identify the main areas for improvement.

#### Structured feedback on the original report

The second stage was a round of structured feedback on the original report in October 2022. We sent out a copy of the CanRisk report (produced for the think aloud session) to our network of healthcare professionals working in clinical genetics and primary care, and patient/public partners from the CRUK funded CanRisk programme (PPRPGM-Nov20\100002). The report was split into 12 sections (Table 1) and participants were asked to complete a feedback form in Qualtrics. Participants assigned a degree of relevance to the different sections, providing a separate score for both individuals undergoing the risk assessment and healthcare professionals. Participants were asked about whether having two reports, one for individuals undergoing the risk assessment and one for healthcare professionals, or one report for all stakeholders would be better. The form also included short open text questions for participants to expand on their answers and suggest ways of improving the report.

**Table 1:** Original report sections

	**Section title**	**Brief description of content**
**Section 1**	Breast and ovarian cancer model	Table with risk factor categories and gene mutation frequency and sensitivity
**Section 2**	Pedigree	A visual representation of the family history (family tree)
**Section 3**	Summary of genetic tests and pathology	Table with details of genetic tests and pathology in the family
**Section 4**	Summary of age and family cancer diagnoses	Table with details about the age of family members when diagnosed with cancer
**Section 5**	Risk Category (NICE)	Presents the risk assessment results according to NICE categories: population level risk, moderate risk or high risk (text)
**Section 6**	Recommendations for managing risk of breast cancer	Further details about personal risk of developing breast cancer from the age of 20 and between 40 and 50 years compared to the population (text, table and graph)
**Section 7**	Recommendations for risk of carrying a pathogenic gene mutation	NICE guidelines threshold for genetic testing (text)
**Section 8**	Absolute risk of breast and ovarian cancer from current age	Details about the personal absolute risk of developing breast cancer in the next 5 years, 10 years and till age 80 compared to the population (text and table)
**Section 9**	Personal risk of developing breast cancer compared to the population	Graph comparing the personal risk and population level risk of developing breast cancer from current age till age 80
**Section 10**	Breast cancer 10-year risk	Table of 10-year breast cancer risks based on the guidelines contained in the NHS protocol for surveillance of women at very high risk of developing breast cancer
**Section 11**	Mutation carrier probability	List of the probability (%) of having a pathogenic mutation in relevant genes (per specific gene and all relevant genes together)
**Section 12**	Extra information	Details of missing information that could make the calculation more personalised

#### Literature review

While collecting data in step 2, we also conducted a targeted review of the literature on how best to communicate genetic tests results. After a targeted search of key words, relevant titles and abstracts were screened for relevance. A total of eight papers were reviewed in full(Brockman et al. 2021; Dorschner et al. 2014; Farmer et al. 2020; Haga et al. 2014; Lewis et al. 2022; Recchia et al. 2020, 2022; Stuckey et al. 2015), looking for overall recommendations and consistency between the evidence.

#### Development of a new report protype

Based on the results from the feedback from steps 1 and 2, as well as key recommendations from the literature in step 3, we designed a new report prototype. During a period of about 2 months, our multidisciplinary team of researchers met fortnightly to refine and agree on the overall structure and content of the prototype and commented on written progress. Collaboration across our team was key to design a prototype that was effective in terms of the communication strategies, achievable in terms of the web development work, and accurate in terms of the risk prediction model. We focused on presenting the prototype in a format that facilitated asking stakeholders for their feedback. We created a ‘mock’ report using Microsoft Word (Appendix 2), with a focus on the overall structure, signposting, images, tables, graphs, keys and explanatory text supporting the interpretation. We used the comments tool to specify changes for the dynamic elements of the report, namely, omissions and alternative text for cases where information reported varies because of individual variables (e.g., history of contralateral breast cancer) and the risk outcomes (i.e., near population, moderate and high risk).

#### First round of structured feedback on the prototype

In February 2023, we sent the prototype to the same network of stakeholders who were asked to complete a structured feedback form in step 2 supported by Qualtrics. This form checked the clarity of the aim and audience for each section; the clarity and overall accessibility of the text and images (graphs/icon arrays); and the overall signposting and coherence of the prototype by providing statements about each element in the report and asking participants to state their level of agreement on a 5-point scale (1, strongly disagree; 5, strongly agree). The form also included short open text boxes for participants to expand on their evaluation of each section and the report as a whole. The form also asked participants their overall opinion of the report and general likes and dislikes.

#### Updating the new report prototype

After analysing the feedback and prioritising recommendations, we updated the prototype, wrote the web and report specification documents, and commenced development work on the CanRisk tool website to generate the new report. The development work involved refactoring the code for the existing web results page to match the new specifications. The text and images from the web results were used in generating the new CanRisk report.

#### Second round of structured feedback on the prototype

In September 2023, we shared the refined prototype produced by the CanRisk tool with the stakeholders. The same Qualtrics form as in step 5 of the co-design process was used to collect feedback.

#### Finalising and publishing the new CanRisk report

In the final stage, we updated any remaining minor issues identified, finalised the new report and completed the development of the web page and report. Further in-house manual and automated testing of the new results and report was carried out. This was important as the report generation needs to dynamically produce different output in multiple scenarios, e.g. contralateral breast cancer risks, male probands. The new web templates were marked-up to enable translation of the new CanRisk results page and report into French, Dutch, German, Portuguese, Italian and Spanish.

### Data analysis

Qualitative data from the think aloud testing session and the open text box questions in the structured feedback were analysed using content analysis. Quantitative data from the scoring of sections in the report were analysed using descriptive statistics (e.g., percentages, frequency distributions, standard deviations) and parametric tests (ANOVA). The Likert scale of relevance (not at all important – extremely important) from step 2 of the co-design process was converted into a 0—4 scoring system with 4 being the highest level of importance. Each section of the report was then assigned an average score based on all responses.

## Results

In total, 34 people contributed to this co-design process (Table 2). We received 56 sets of feedback over a 13-month period, as some people participated in multiple stages (Table 3).

**Table 2:** Participants

	*Number*
**Think-aloud session (step 1)**	
*Background*	
Public partner	3 (50%)
Patient partner	3 (50%)
*Gender*	
Female	5 (83%)
Male	1 (17%)
*Sub-total*	*6*
**Co-design process (steps 2–8)**	
*Background*	
Public partner	12 (38.7%)
Primary Care Healthcare Professional	10 (32.2%)
Clinical Genetics Healthcare Professional	6 (19.4%)
Patient partner	3 (9.7%)
*Gender*	
Female	26 (83.9%)
Male	5 (16.1%)
*Sub-total*	*31*

*Note: 3 of the 6 people who were involved in the think-aloud session (step 1) continued to contribute co-design process, which is why the total of participants is 34 (steps 2–8)

**Table 3:** Feedback iterations

	Think-aloud session (original report) (September 2022)	Original report feedback (October 2022)	New report prototype feedback 1 (February 2023)	New report prototype feedback 2 (September 2023)	***Total***
Stakeholder					
Public Partners	3	3	2	9	**17**
Primary Care Healthcare Professional	-	7	4	6	**17**
Clinical Genetics Healthcare Professional	-	4	4	4	**12**
Patient Partners	3	3	3	1	**10**
***Total***	**6**	**17**	**13**	**20**	**56**

### Think-aloud testing of the original CanRisk report

During the think aloud testing session, participants reported being overwhelmed by the amount of information presented in the report, the medical jargon, tables and graphs, and the lack of structure and explanatory text. Examples notes from a range of patient/public partners participating in this activity include:

*“What am I meant to do with all of this?”; “Am I meant to read all of this?”; “What are all these genes? What do they mean?”; “How do I read this?”; “What is ‘PRS’?”; “What is tubal ligation?”*

Further issues identified in this activity included that participants expected the report to start by telling them their specific results and although the NICE Risk Category section was helpful for this aim, it was presented later in the report. Participants found the graphs and mathematical symbols difficult to understand; the polygenic scores, genetic tests and tumour pathology information were particularly challenging. Participants expressed interest in this information but required support to interpret and understand it. They also stressed the importance of the report being clear about addressing the individual undertaking the risk assessment, having a consistent audience (i.e. avoid sometimes addressing clinicians and sometimes addressing individuals undergoing the assessment) and avoiding indirect forms of speech (i.e. ‘the’ risk versus ‘your’ risk). Participants highlighted the need for more clarity when comparing ‘personal risk’ and ‘population risk’. Formatting issues identified included making sure graphs worked in black and white, a preference for smaller scale icon arrays (i.e., 100 instead of 1000) and adding headings and keys for all graphs and tables.

This activity allowed us to establish that the original CanRisk report was not accessible for patients and members of the public and confirmed the need to engage with a co-design process to develop a new report.

### Structured feedback on the original report

Seventeen stakeholders from a network of healthcare professionals working in clinical genetics and primary care, and our existing network of patient/public partners provided feedback on the same report tested in the think-aloud session (Table 3). Participants scored sections 1–12 in the report by their relevance for both individuals undergoing the risk assessment and healthcare professionals (see Table 4). The average scores showed that half of the sections (sections 1-4 and 11–12) were more relevant (i.e. scored 2 or above on average) for healthcare professionals than individuals undergoing the risk assessment. The remaining half (sections 5–10) scored highly for individuals undergoing the risk assessment and healthcare professionals. The scores relating to the importance of each section for individuals undergoing the risk assessment did not differ between groups of respondents for 11 out of 12 sections. For Sect. 3 (Summary of genetic tests and pathology), there was a significant difference (F(5,11) = 3.42, *p* < 0.05). Similarly, the scores relating to the importance of each section for healthcare professionals did not differ between groups of respondents for 11 out of 12 sections. For Sect. 11 (Mutation carrier probability), there was a significant difference (F(5,11) = 7.42, *p* < 0.01).

**Table 4:** Relevance per report section for different audiences

		Section importance for individuals undergoing the risk assessment	Section importance for healthcare professionals
		Mean (SD)	Mean (SD)
Section 1: Breast and ovarian cancer model	Public (*n* = 5)	2.80 (1.10)	3.00 (1.00)
	Clinical Geneticist (*n* = 2)	1.50 (0.71)	2.50 (1.71)
	Other (*n* = 3)	2.33 (2.08)	3.00 (1.00)
	GP (*n* = 4)	0.75 (0.50)	2.50 (0.58)
	Genetic counsellor (*n* = 2)	1.00 (0.00)	3.00 (0.00)
	Practice Nurse (*n* = 1)	*	4.00 (0.00)
	Total	1.70 (1.36)	2.87 (0.74)
Section 2: Pedigree	Public (*n* = 5)	2.60 (1.14)	3.67 (0.58)
	Clinical Geneticist (*n* = 2)	2.50 (0.71)	3.00 (1.41)
	Other (*n* = 3)	2.00 (1.00)	3.00 (0.00)
	GP (*n* = 4)	1.25 (0.05)	3.00 (0.00)
	Genetic counsellor (*n* = 2)	2.00 (0.00)	3.00 (0.00)
	Practice Nurse (*n* = 1)	*	2.00 (0.00)
	Total	1.94 (1.03)	3.07 (0.59)
Section 3: Summary of genetic tests and pathology	Public (*n* = 5)	3.00 (1.23)	3.67 (0.58)
	Clinical Geneticist (*n* = 2)	1.00 (1.41)	3.00 (1.41)
	Other (*n* = 3)	1.33 (1.53)	2.00 (1.73)
	GP (*n* = 4)	0.25 (0.50)	1.50 (0.58)
	Genetic counsellor (*n* = 2)	0.50 (0.71)	2.50 (0.71)
	Practice Nurse (*n* = 1)	*	2.00 (0.00)
	Total	1.35 (1.50)	2.40 (1.18)
Section 4: Summary of age and family cancer diagnoses	Public (*n* = 5)	2.80 (1.30)	3.33 (1.16)
	Clinical Geneticist (*n* = 2)	1.00 (1.41)	2.50 (0.71)
	Other (*n* = 3)	2.67 (2.31)	2.00 (1.73)
	GP (*n* = 4)	0.75 (0.96)	2.25 (0.96)
	Genetic counsellor (*n* = 2)	*	2.00 (0.00)
	Practice Nurse (*n* = 1)	*	2.00 (0.00)
	Total	1.58 (1.66)	2.40 (1.06)
Section 5: Risk Category (NICE)	Public (*n* = 5)	3.20 (0.84)	3.33 (1.1.6)
	Clinical Geneticist (*n* = 2)	2.50 (0.71)	3.50 (0.71)
	Other (*n* = 3)	3.33 (0.58)	3.67 (0.58)
	GP (*n* = 4)	3.25 (1.50)	3.50 (0.58)
	Genetic counsellor (*n* = 2)	2.50 (0.71)	3.00 (1.41)
	Practice Nurse (*n* = 1)	4.00 (0.00)	4.00 (0.00)
	Total	3.12 (0.93)	3.47 (0.74)
Section 6: Recommendations for managing risk of breast cancer	Public (*n* = 5)	2.80 (1.30)	3.67 (0.58)
	Clinical Geneticist (*n* = 2)	2.50 (0.71)	3.00 (1.41)
	Other (*n* = 3)	2.67 (0.58)	3.33 (0.58)
	GP (*n* = 4)	2.75 (0.96)	3.75 (0.50)
	Genetic counsellor (*n* = 2)	3.00 (1.41)	4.00 (0.00)
	Practice Nurse (*n* = 1)	4.00 (0.00)	4.00 (0.00)
	Total	2.82 (0.95)	3.60 (0.63)
Section 7: Recommendations for risk of carrying a pathogenic gene mutation	Public (*n* = 5)	2.60 (1.34)	3.00 (1.73)
	Clinical Geneticist (*n* = 2)	2.50 (0.71)	3.50 (0.71)
	Other (*n* = 3)	2.33 (1.16)	3.33 (0.58)
	GP (*n* = 4)	2.25 (1.26)	3.25 (0.50)
	Genetic counsellor (*n* = 2)	2.50 (0.71)	2.50 (2.12)
	Practice Nurse (*n* = 1)	4.00 (0.00)	4.00 (0.00)
	Total	2.53 (1.07)	3.20 (1.01)
Section 8: Absolute risk of breast and ovarian cancer from current age	Public (*n* = 5)	2.60 (1.34)	3.00 (1.73)
	Clinical Geneticist (*n* = 2)	2.50 (0.71)	3.00 (1.41)
	Other (*n* = 3)	2.00 (0.00)	3.33 (0.58)
	GP (*n* = 4)	1.75 (1.23)	2.75 (0.96)
	Genetic counsellor (*n* = 2)	3.50 (0.71)	3.50 (0.71)
	Practice Nurse (*n* = 1)	4.00 (0.00)	4.00 (0.00)
	Total	2.47 (1.13)	3.13 (0.99)
Section 9: Personal risk of developing breast cancer compared to the population	Public (*n* = 5)	2.00 (1.23)	3.00 (1.73)
	Clinical Geneticist (*n* = 2)	3.00 (0.00)	2.50 (0.71)
	Other (*n* = 3)	1.67 (0.58)	3.33 (0.58)
	GP (*n* = 4)	2.25 (0.56)	3.25 (0.50)
	Genetic counsellor (*n* = 2)	3.00 (0.00)	2.50 (2.12)
	Practice Nurse (*n* = 1)	4.00 (0.00)	4.00 (0.00)
	Total	2.35 (1.00)	3.07 (1.03)
Section 10: Breast cancer 10-year risk	Public (*n* = 5)	2.60 (0.89)	2.67 (1.53)
	Clinical Geneticist (*n* = 2)	3.50 (0.71)	2.00 (0.00)
	Other (*n* = 3)	2.33 (0.58)	2.67 (0.58)
	GP (*n* = 4)	2.75 (0.50)	2.75 (1.23)
	Genetic counsellor (*n* = 2)	4.00 (0.00)	1.00 (0.00)
	Practice Nurse (*n* = 1)	4.00 (0.00)	4.00 (0.00)
	Total	2.94 (0.83)	2.47 (1.13)
Section 11: Mutation carrier probability	Public (*n* = 5)	2.60 (1.95)	3.33 (1.16)
	Clinical Geneticist (*n* = 2)	2.00 (0.00)	4.00 (0.00)
	Other (*n* = 3)	2.67 (0.58)	3.33 (0.58)
	GP (*n* = 4)	0.25 (0.50)	1.25 (0.50)
	Genetic counsellor (*n* = 2)	2.50 (1.71)	4.00 (0.00)
	Practice Nurse (*n* = 1)	2.00 (0.00)	3.00 (0.00
	Total	1.94 (1.44)	2.93 (1.22)
Section 12: Extra information	Public (*n* = 5)	1.60 (1.52)	3.67 (0.58)
	Clinical Geneticist (*n* = 2)	*	3.00 (1.41)
	Other (*n* = 3)	2.00 (1.73)	2.33 (1.16)
	GP (*n* = 4)	0.25 (0.50)	1.00 (0.00)
	Genetic counsellor (*n* = 2)	0.50 (0.71)	2.00 (1.41)
	Practice Nurse (*n* = 1)	*	2.00 (0.00)
	Total	0.94 (1.30)	2.27 (1.22)

Note: *denotes missing information

Feedback was split in terms of whether it would be better to have one or two reports, with eight participants preferring one report for all audiences and nine preferring separate reports for individuals undergoing the risk assessment and healthcare professionals. Open text box answers highlighted the importance of including as much information as possible while keeping it as simple as possible. The main suggestions on how to achieve this included having a summary with key information for individuals undergoing the risk assessment at the start of the report, using lay terms, merging sections that covered similar information, adding definitions when relevant and explanatory text to support interpretation, and signposting so different stakeholders can go straight to the information most relevant to them.

### Literature review

The review of eight relevant papers (Brockman et al. 2021; Dorschner et al. 2014; Farmer et al. 2020; Haga et al. 2014; Lewis et al. 2022; Recchia et al. 2020, 2022; Stuckey et al. 2015) informed decision making around three key areas and where consensus was not reached from participants; the format of the report, the flow of information, and the content.

*Report format:*

Stuckey et al. (2015) suggested that having one document is better to ensure that everyone receives the same information. Their recommendation to manage large amounts of information is to split reports into sections and use signposting. Recchia et al. (2022) showed that output reports from high risk genes tests for breast cancer should focus on what the results mean for the individuals undergoing the assessment while also supporting clinicians in the appropriate management.

In light of this, we agreed to take a ‘one report’ approach, which addresses the individual undergoing the risk assessment and starts with a section covering the results that are most relevant and informative to them. The remaining sections of the report are consistently addressed to the individual undergoing the assessment but are introduced as potentially more relevant for healthcare professionals and provide the technical information clinicians need to discuss management options across different levels of care (i.e., referral to specialist care, further testing, etc.).

*Report flow:*

Research by Brockman et al. (Brockman et al. 2021) indicated that reports perform better if they can adapt to stakeholders needs and context, as people report different levels of interest in complex information as well as different preferences in risk presentation formats. To address this as much as possible, and in addition to presenting risk in different formats, we wanted the CanRisk report to be ‘dynamic’, covering the content that is relevant for specific personal and risk factor information entered into the tool (e.g. for male probands and those with contralateral breast cancer).

*Report content:*

Some of the more straightforward recommendations from the literature in terms of the content of reports included a preference for absolute risk (actual probability of an outcome) across stakeholders (Farmer et al. 2020; Lewis et al. 2022) and using the word ‘pathogenic variant’ over ‘mutation’ when presenting or discussing genetic information (Stuckey et al. 2015). These recommendations aimed to increase clarity and confidence in individuals undergoing risk assessments and were either already in the original report (absolute risk) or adopted for the new CanRisk report (variant) to improve its communication quality.

### Development of a new report prototype

Based on results from steps 1, 2, and 3, the new report prototype included an introductory page to facilitate navigating the report. The information in the report was split into three sections, where section one compiled the information that is most relevant for the individual undergoing the assessment, and sections two and three compiled information that may be most relevant for healthcare professionals with increasing level of complexity. More details about the new report prototype are shown in Table 5.

**Table 5:** Structure of new report prototype

**Introduction**	**Section 1:** ‘Information relevant for you’	Section 2*: ‘Information more relevant for healthcare professionals’	Section 3*: ‘Information more relevant for specialist healthcare professionals’
• Bullet point list of the information included in the report • Specification of what information may be most relevant for different stakeholders • Setting expectations regarding clinical recommendations. People should contact their usual healthcare provider if they wish to discuss how to manage or reduce their risk of developing cancer	Risk calculation results using explanatory text, definitions, icon arrays, graphs and the NICE risk categories to support the interpretation of the information	More detailed information about: • The individual’s future risk of developing cancer according to the NICE guidelines • Individual risk of developing cancer between now and the age of 80 years compared to the population risk over the same time • The estimated risk of carrying an inherited pathogenic gene variant	• Visual representation of the family history (family tree or pedigree) • Summary of genetic tests and pathology; the cancer models including risk factor details and pathogenic variant frequencies and test sensitivity • Polygenic scores • Summary of cancer diagnoses in the family • Advisory notes

*Sections two and three also included explanatory text, definitions, graphs, keys and tables to support the interpretation of the information

### First round of structured feedback on the prototype

Thirteen people provided feedback on the initial prototype (Table 3). Overall, three (23%) people ranked this prototype as ‘excellent’ and ten (77%) as ‘good’. Most responses either ‘strongly agreed’ or ‘somewhat agreed’ with positive statements about the clarity, aim, relevance, and accessibility of each element in the report as well as the report’s overall signposting and cohesiveness. The structured feedback included a total of thirty-one statements (Table 6). Where elements received two or more (≥ 15%) neutral or negative evaluations (9/31, 29%), we reviewed these (bold text in Table 6) alongside the results from the open text boxes. This qualitative feedback provided recommendations on how to make the front page clearer and more concise by reordering certain content, simplifying text entries, and making the narrative voice consistent.

**Table 6:** Structured feedback questionnaire responses from round 1 (n = 13)

**Section**	**Statement**	**Somewhat/Strongly agree**	**Neither agree nor disagree**	**Somewhat/Strongly disagree**	Blank/missing data
**Front page:** Introduction and overview of the report	*“It is clear from the introduction that the report is primarily addressed to the individual undergoing the risk assessment”*	11 (84%)	*1 (8%)*	0 (0%)	1 (8%)
	*“The introduction is easy to understand”*	11 (84%)	*1 (8%)*	0 (0%)	1 (8%)
	*“The information presented in the introduction is important”*	12 (92%)	0 (0%)	0 (0%)	1 (8%)
	*“The introduction presents a good overview of the report”*	12 (92%)	0 (0%)	0 (0%)	1 (8%)
	*“The introduction helps the individual undergoing the risk assessments and healthcare professionals navigate the report”*	12 (92%)	0 (0%)	0 (0%)	1 (8%)
	*“The introduction sets clear expectations from the report”*	11 (84%)	0 (0%)	0 (0%)	2 (15%)
Section 1: Information relevant for individuals undergoing the risk assessment	*“Sect. 1 as a whole is clear and relevant, especially for the individual undergoing the risk assessment”*	12 (92%)	0 (0%)	0 (0%)	1 (8%)
	*“This section clearly explained how risk can change over the next 5 years, 10 years and by age 80”*	12 (92%)	0 (0%)	0 (0%)	1 (8%)
	*“The text explaining the individual’s risks of developing breast and or ovarian cancer is clear and concise”*	12 (92%)	0 (0%)	0 (0%)	1 (8%)
	*“The images with the 100 dots help understand and visualise the risk results presented in percentages and text”*	11 (84%)	*1 (8%)*	0 (0%)	1 (8%)
	*“This section clearly explains the difference between personal risk and population risk”*	11 (84%)	*1 (8%)*	0 (0%)	1 (8%)
	*“Text explanations help understand the graph showing the individual’s risk of developing breast and or ovarian cancer relative to the population risk”*	12 (92%)	0 (0%)	0 (0%)	1 (8%)
	*“This section clearly presents the individual’s risk category according to the NICE guidelines”*	11 (84%)	*1 (8%)*	0 (0%)	1 (8%)
	*“Notes are clear and helpful”*	11 (84%)	*1 (8%)*	0 (0%)	1 (8%)
Section 2: Information more relevant for healthcare professionals	*“Sect. 2 as a whole is clear and relevant for the individual and healthcare professionals”*	10 (77%)	*1 (8%)*	0 (0%)	2 (15%)
	*“The first statement helps understand how the individual’s NICE risk category is defined”*	11 (84%)	*1 (8%)*	0 (0%)	1 (8%)
	*“This section clearly presents the individual’s risk between the ages of 20 and 80 years of developing breast cancer”*	10 (77%)	0 (0%)	0 (0%)	3 (23%)
	*“This section clearly presents the individual’s risk of developing breast cancer specifically between the ages of 40 and 50”*	12 (92%)	0 (0%)	0 (0%)	1 (8%)
	***“The table presenting the ranges for the NICE risk categories is adequately introduced and it helps understanding how the categories work”***	9 (69%)	**3 (23%)**	0 (0%)	1 (8%)
	***“The graph showing the individual’s NICE risk categories compared to the population is clearly explained and helpful to understanding the individual’s risks”***	8 (62%)	**2 (15%)**	**2 (15%)**	1 (8%)
	*“The tables showing how the individual’s risks of developing breast and or ovarian cancer changes over time compared to the population is clear”*	11 (84%)	0 (0%)	*1 (8%)*	1 (8%)
	***“The risk of carrying a genetic pathogenic variant is clearly explained and the results easy to find”***	6 (46%)	**6 (46%)**	0 (0%)	1 (8%)
Section 3: Information more relevant for specialist healthcare professionals	***“Sect. 3 as whole is clear and relevant, especially for healthcare professionals”***	9 (69%)	**1 (8%)**	**2 (15%)**	1 (8%)
	***“The family pedigree is clearly introduced”***	8 (62%)	**3 (23%)**	**1 (8%)**	1 (8%)
	***“The summary of genetic tests was clearly introduced and the note below it helps understand the table”***	7 (54%)	**2 (15%)**	**3 (23%)**	1 (8%)
	***“It is clear that the breast and or ovarian cancer model tables summarised the information used to calculate the individual's risk of breast and or ovarian cancer”***	10 (77%)	**1 (8%)**	**1 (8%)**	1 (8%)
	*“The breast and or ovarian cancer polygenic scores are clearly explained and the results adequately presented relative to the population”*	12 (92%)	0 (0%)	0 (0%)	1 (8%)
	*“The graph showing the distribution of polygenic scores in the population helps understand and visualise the results presented in percentages and text”*	11 (84%)	0 (0%)	1 (8%)	1 (8%)
	*“The bar figure presenting ‘low’ and ‘high’ polygenic scores helps understand and visualise the results presented in percentages and text”*	12 (92%)	0 (0%)	0 (0%)	1 (8%)

### Updating the new report prototype

Following the initial feedback on the prototype, we added information about how data were used to calculate the risk score, simplified text entries and word choices (i.e., ‘image’ over ‘icon’) and replaced ‘lifetime risk’ with ‘risk between the ages of 20 and 80’. We also changed the order of the information on genetic pathogenic variant probabilities to present general and positive information first and then introduce more detailed and complex information; explained what a ‘family tree’ is and added further details on the information used to make it; expanded the key (list instead of paragraph) for the summary of genetic tests and cancer diagnoses; explained that risk factors included in the model can increase or decrease risk; and reduced the amount of information and changed the presentation format (table instead of paragraph) of the ‘extra information’.

### Second round of structured feedback on the prototype

Twenty people provided feedback on the revised prototype (Appendix 3). Eleven (55%) participants scored this prototype as ‘excellent’, eight as ‘good’ (40%) and one (5%) as ‘average’, indicating improved performance from the earlier prototype. Most participants either ‘strongly agreed’ or ‘somewhat agreed’ with positive statements about the clarity, aim, relevance, and accessibility of each element in the report as well as the report’s overall signposting and cohesiveness. Table 7 shows all results for the twenty-one elements (out of thirty-one as shown in Table 6) that received neutral or somewhat negative evaluations. Where elements received three or more (≥ 15%) neutral or negative evaluations (5/31, 16%) we reviewed these (bold text in Table 7) alongside the results from the open text boxes. This qualitative feedback helped us identify formatting errors, adjust the size and contrast of images and text, and improve signposting by making section headings clearer. Some of their recommendations helped us simplify text entries and improve explanations. We received several pieces of spontaneous encouraging feedback – for example: *“I would have liked the information presented in that way when I was given my risk factor”.* (Table 7)

**Table 7:** Statements with neutral and or somewhat negative evaluations from round 2 of structured feedback (n = 20)

**Section**	**Statement**	**Somewhat/Strongly agree**	**Neither agree nor disagree**	**Somewhat/Strongly disagree**	Blank/missing data*
Front page: Introduction and overview of the report	*“The front page is easy to understand”*	18 (90%)	*1 (5%)*	0 (0%)	1 (5%)
	*“Information presented in the front page is important”*	18 (90%)	*1 (5%)*	0 (0%)	1 (5%)
	*“The front page presents a good overview of the report”*	18 (90%)	*1 (5%)*	0 (0%)	1 (5%)
Section 1: Information relevant for individuals	*“Text explanations help understand the graph showing the individual’s risk of developing breast and ovarian cancer relative to population risk”*	18 (90%)	*1 (5%)*	0 (0%)	1 (5%)
	*“This section clearly presents the individual’s risk category according to the NICE guidelines”*	17 (85%)	*2 (10%)*	0 (0%)	1 (5%)
	*“Notes are clear and helpful”*	17 (85%)	*2 (10%)*	0 (0%)	1 (5%)
Section 2: Information more relevant for healthcare professionals	*“Sect. 2 as a whole is clear and relevant for the individual and healthcare professionals”*	16 (80%)	0 (0%)	*2 (10%)*	2 (10%)
	*“The first statement helps understand how the individual’s NICE risk category is defined”*	17 (85%)	0 (0%)	*2 (10%)*	1 (5%)
	*“This section clearly presents the individual’s risk between the ages of 20 and 80 of developing breast cancer”*	17 (85%)	0 (0%)	*2 (10%)*	1 (5%)
	*“This section clearly presents the individual’s risk of developing breast cancer specifically between the ages of 40 and 50”*	17 (85%)	0 (0%)	*2 (10%)*	1 (5%)
	***“The table presenting the ranges for the NICE risk categories is adequately introduced and it helps understanding how the categories work”***	14 (70%)	**2 (10%)**	**3 (15%)**	1 (5%)
	***“The graph showing the NICE risk categories of the individual compared to the population is clearly explained and helpful to understanding the individual’s risks”***	13 (65%)	**2 (10%)**	**4 (20%)**	1 (5%)
	*“The tables showing how the individual’s risks of developing breast and or ovarian cancer changes over time compared to the population is clear”*	17 (85%)	0 (0%)	1 (5%)	2 (10%)
	***“The risk of carrying a genetic pathogenic variant is clearly explained and the results easy to find”***	16 (80%)	**2 (10%)**	1 (5%)	1 (5%)
Section 3: Information more relevant for specialist healthcare professionals	*“The summary of genetic tests was clearly introduced and the note below it helps understand the table”*	18 (90%)	*1 (5%)*	0 (0%)	1 (5%)
	***“It is clear that the breast and or ovarian cancer model tables summarised the information used to calculate the risk of breast and or ovarian cancer of the individual”***	16 (80%)	**2 (10%)**	**1 (5%)**	1 (5%)
	*“The breast and ovarian cancer polygenic scores are clearly explained, and the results adequately presented relative to the population”*	18 (90%)	*1 (5%)*	0 (0%)	1 (5%)
	*“The graph showing the distribution of polygenic scores in the population helps understand and visualise the results presented in percentages and text”*	17 (85%)	*2 (10%)*	0 (0%)	1 (5%)
	*“The bar figure presenting ‘low’ and ‘high’ polygenic scores helps understand and visualise the results presented in percentages and text”*	18 (90%)	*1 (5%)*	0 (0%)	1 (5%)
	*“The summary of cancer diagnoses in the family of the individual undergoing the risk assessment is clearly introduced and the note below it helps understand the table”*	17 (85%)	0 (0%)	*2 (10%)*	1 (5%)
	***“The advisory note section is clear”***	16 (80%)	**3 (15%)**	0 (0%)	1 (5%)

### Finalising and publishing the new CanRisk report

The main changes incorporated in the final report included further improving formatting issues (i.e., font and image size, bold text) and simplifying text, particularly around the NICE risk categories. We also removed unnecessary details about the ‘average population’; added details about the NICE guideline referenced; fixed a format problem with the NICE categories table; and replaced the graph showing the NICE risk categories of individual undergoing the risk assessment compared to the population.

Although patient/public partners and healthcare professionals (supported by previous research (Farmer et al. 2020; Recchia et al. 2022)) were keen for the report to include ‘next steps’ for clinicians and more information about what people can expect in the future, it was decided that adding this information to the new CanRisk report was not consistent with the tool’s intended purpose. Furthermore, the international nature of the CanRisk tool would make adding this difficult, since management recommendations can vary from country to country and, sometimes, between regions. After completing the development of the last changes, the broader research team reviewed the protype and we decided to draw the co-design process to a close and finalise the report. Using current web technologies, the new report PDF is produced within the web browser and not on the server. The reports are dynamically customised based on the user’s input by tailoring the explanatory introduction and NICE guidelines text, excluding any optional sections, showing relevant reference population risks, and suggesting how the risk predictions might be improved with more data. The CanRisk website and the new report are available in French, Dutch, German, Portuguese, Italian and Spanish. To ensure readability and aesthetic layout the text was formatted for all possible risk calculation scenarios and languages. The newly designed CanRisk report is available at www.canrisk.org; see appendix 4 for a moderate risk example of the report in English.

## Discussion

As multifactorial cancer risk prediction tools such as CanRisk are increasingly being incorporated into routine healthcare, the effective presentation of risk information for individuals undergoing the risk assessments and healthcare professionals is key for effective implementation (Brigden et al. 2023; Goldman et al. 2006; Julian-Reynier et al. 2003). Our aim was to determine how to best present the results from the CanRisk tool and produce a new report that presents key information for individuals undergoing the risk assessment and better supports healthcare professionals to communicate risk effectively. This paper presents an 8-step co-design process to develop the new CanRisk report through a collaboration between CanRisk researchers, patients, members of the public and healthcare professionals. Through this process, we established that the original CanRisk report was not suitable for patients and members of the public and that healthcare professionals working in different settings would primarily focus on different information (e.g. individual risk category compared to the population in primary care contexts versus pedigree information in clinical genetics). We were subsequently able to identify the needs of different user groups, prioritise recommendations from the literature, and guide the development and update of the new report prototype.

The co-design approach was particularly effective in increasing the diversity of ideas involved in developing a solution to the report format and reducing the development and maintenance costs of the final report (Vargas et al. 2022). Results around the relevance of the different elements in the report for patients, public members and healthcare professionals were central to identifying a clear and logical organisation of the information in the report. This helped improve the quality of the report and the user experience (Vargas et al. 2022) as it is key to support understanding of complex information (Stuckey et al. 2015). The final two iterations of feedback continued to contribute toward the quality and user experience of the prototype, while also ensuring the reduction of error (Vargas et al. 2022). In this final stage, the co-design process helped ensure that we were developing a report that is visually appealing, easy to navigate and uses accessible language. Finally, the ‘dynamic’ feature of the new CanRisk report means that the level of information and complexity is bespoke to the individual risk assessment, enhancing the users’ experience. In practice, this also means that some information is optional, such as the NICE risk categories (which are not relevant for international users); ovarian cancer risk; and advisory notes, and that the content varies slightly depending on the clinical history of the individual undergoing the assessment.

Completing a co-design process was central to successfully achieving our aim. This collaborative and iterative process allowed for the breadth of issues identified and addressed to go beyond those identified in previous research; it encouraged the research team to maintain a reflexive attitude throughout the process; and ensured that the outcome is relevant for a broad range of stakeholders (patients, members of the public and healthcare professionals working in primary care and clinical genetics). Still, this approach was resource intensive. The time taken to complete the number of iterations required to achieve the desired outcome meant that our broader research programme and outcomes needed to be reorganised slightly. Further, sustaining an iterative process over a long period of time can have an impact on the engagement/availability of members within the stakeholder group across the study that may, in part, explain the variable number of inputs we had at each stage. This approach also required significant time and coordination skills to manage the needs of a multidisciplinary research team as well as those of the group of stakeholders. Managing continuous and relevant communications with researchers and members of the stakeholder group was crucial to facilitating collaboration, ensuring everyone could complete their tasks in a timely manner, and preventing delays at each stage. Anticipating and planning accordingly could improve the experience and outcome of future researchers interested in adopting a similar approach.

In the future, we aim to explore differences in perceived risk, knowledge of breast cancer risk, comprehension, and communication efficacy of the new CanRisk report compared to the original CanRisk report. We also plan to expand the new CanRisk report to include the displaying of the results for other cancer risk assessments (e.g., prostate cancer).

## Conclusion

This research shows the value of collaboration for the successful communication of complex health information and, as a result, the new CanRisk report has the potential to better support shared decision-making processes about the management of cancer risk across clinical settings. Co-design provided a useful framework to find effective ways of communicating multifactorial risk prediction outcomes to different stakeholders. Specifically, engaging in active collaboration with stakeholders allowed us to find creative solutions to address key challenges. Beyond the more granular improvements this brought to the report in terms of how to present and support the interpretation of specific information, the most notable outcome of our collaboration was how researchers, patients, members of the public and healthcare professionals worked together to identify a consistent hierarchy for the risk information that needed to be presented. This defined the main concept for the new CanRisk report (i.e., one report for all stakeholders focusing on the individual undergoing the assessment), its overall structure (i.e., three clearly labelled sections with increasing level of detail and complexity), and how to support different audiences navigating the report. None of this would have been possible using a purely consultative approach focusing solely on previous research (i.e., literature) and/or a heterogenous group of stakeholders without the iterative element that characterises co-production and co-design methodologies.

## Supplementary Information

Below is the link to the electronic supplementary material.

Supplementary file1 (DOCX 622 KB)

Supplementary file2 (DOCX 449 KB)

Supplementary file3 (PDF 452 KB)

Supplementary file4 (PDF 774 KB)

Supplementary file5 (JPG 22 KB)

## Data Availability

Data is provided within the manuscript.
