# Correction: Improving the communication of multifactorial cancer risk assessment results for different audiences: a co-design process

**DOI:** 10.1007/s12687-024-00745-4

**Published:** 2024-10-25

**Authors:** Francisca Stutzin Donoso, Tim Carver, Lorenzo Ficorella, Nichola Fennell, Antonis C. Antoniou, Douglas F. Easton, Marc Tischkowitz, Fiona M. Walter, Juliet A. Usher-Smith, Stephanie Archer

**Affiliations:** 1 Department of Public Health and Primary Care, University of Cambridge, Cambridge, UK; 2 Department of Medical Genetics, National Institute for Health Research Cambridge Biomedical Research Centre, University of Cambridge, Cambridge, UK; 3 Wolfson Institute of Population Health, Barts and the London School of Medicine and Dentistry, Queen Mary University of London, London, UK; 4 Department of Psychology, University of Cambridge, Cambridge, UK

**Journal of Community Genetics**

10.1007/s12687-024-00729-4

In the published original article, the Funding text is missing.

The funding text should be:

This work was supported by Cancer Research UK grant: PPRPGM-Nov20100002.

The original article has been corrected.

