# Supplementary material for: Improving the communication of multifactorial cancer risk assessment results for different audiences: a co-design process
Source: J Community Genet. 2024 Sep 25;15(5):499–515. doi: 10.1007/s12687-024-00729-4 (PMC11549070; doi:10.1007/s12687-024-00729-4)
Supplement: Supplementary file 1 — Supplementary file1 (DOCX 622 KB) [file 12687_2024_729_MOESM1_ESM.docx]

# Breast Cancer Model

| **Risk Factor** | | **Value** |
| --- | --- | --- |
| Age at First Occurrence of Menstruation | | 11 |
| Number of Children | | >2 |
| Age of First Live Birth | | <20 |
| Oral Contraception Usage | | former |
| Hormone Replacement Therapy | | never/former |
| Body Mass Index | | >=30 |
| Alcohol Intake (grams/day) | | 25-<35 |
| Age of Menopause | | - |
| Mammographic Density | | - |
| Height (cm) | | 167.64000000000001 |
| **Gene** | **Mutation frequency** | **Mutation sensitivity** |
| BRCA1 | 0.0006394 | 0.89 |
| BRCA2 | 0.00102 | 0.96 |
| PALB2 | 0.00064 | 0.92 |
| ATM | 0.0018 | 0.94 |
| CHEK2 | 0.00373 | 0.98 |
| BARD1 | 0.00043 | 0.89 |
| RAD51C | 0.00035 | 0.78 |
| RAD51D | 0.00035 | 0.86 |

**Mutation frequency:** UK **Cancer incidence rates:** UK **Please note the model has been developed using data from European ancestry populations.**

**Version:** boadicea model 6.2.0, version 0.6.0; **Timestamp:** 2022-09-29T12:45:33.320202+01:00

# Ovarian Cancer Model

| **Risk Factor** | | **Value** |
| --- | --- | --- |
| Number of Children | | >1 |
| Duration of Oral Contraception Use | | 10-14 |
| Hormone Replacement Therapy | | never |
| Tubal Ligation | | no |
| Endometriosis | | no |
| Body Mass Index | | >=30 |
| Height (cm) | | 167.64000000000001 |
| **Gene** | **Mutation frequency** | **Mutation sensitivity** |
| BRCA1 | 0.0007947 | 0.89 |
| BRCA2 | 0.002576 | 0.96 |
| RAD51D | 0.00035 | 0.86 |
| RAD51C | 0.00035 | 0.78 |
| BRIP1 | 0.00071 | 0.95 |
| PALB2 | 0.00064 | 0.92 |

**Mutation frequency:** UK **Cancer incidence rates:** UK **Please note the model has been developed using data from European ancestry populations.**

**Version:** ovarian model 2.2.0, version 0.6.0; **Timestamp:** 2022-09-29T12:45:33.215309+01:00

**Pedigree**


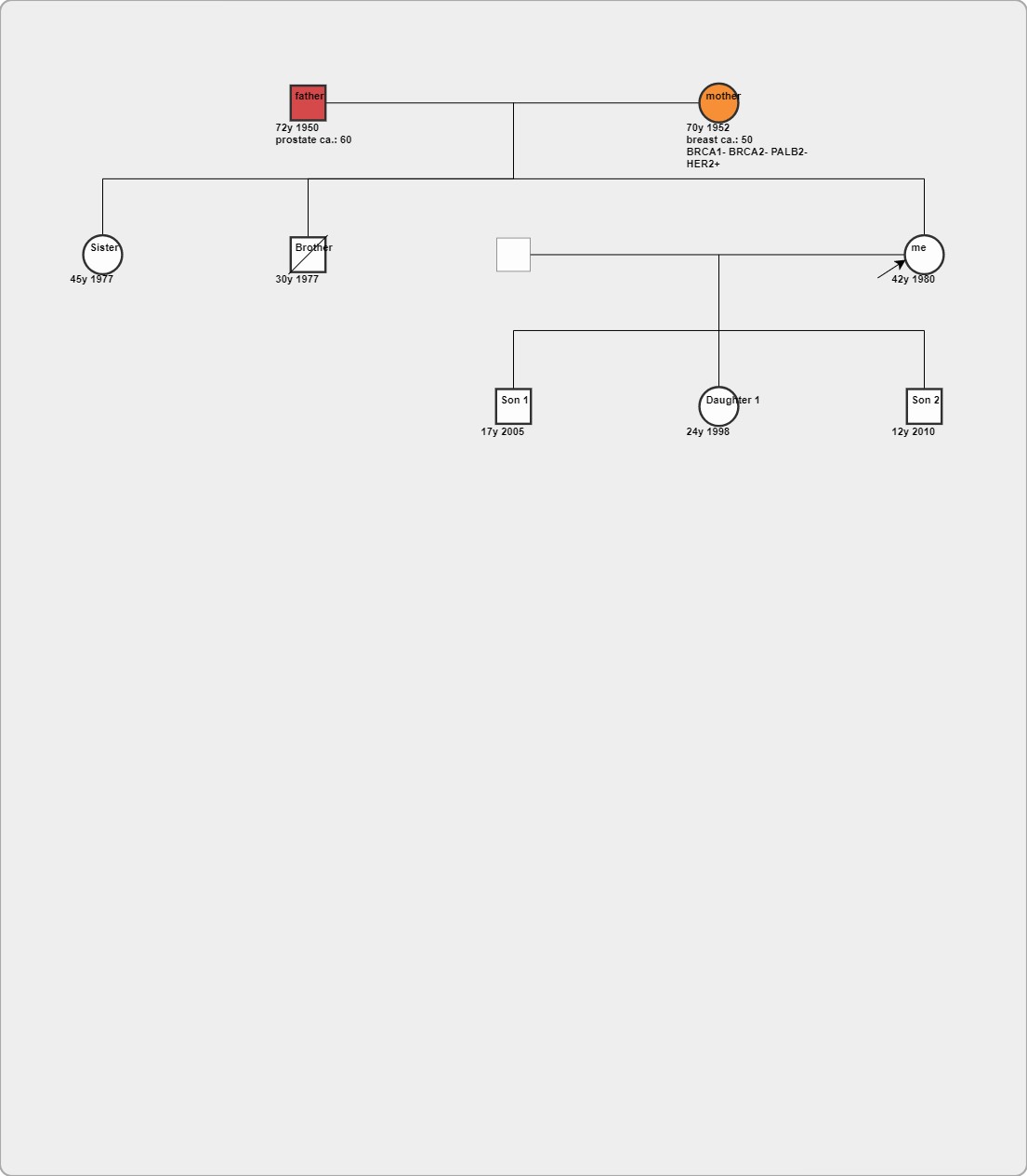


# Summary of Genetic Tests and Pathology

|  |  | **Genetic Tests†** | |  | | |  |  |  |  | **Pathology** |
| --- | --- | --- | --- | --- | --- | --- | --- | --- | --- | --- | --- |
| **Name** |  | **BRCA1** | **BRCA2** | **PALB2** | **ATM** | **CHEK2** | **BARD1** | **RAD51D** | **RAD51C** | **BRIP1** | **ER:PR:HER2:CK14:CK56‡** |
| mother |  | S:N | S:N | S:N | 0:0 | 0:0 | 0:0 | 0:0 | 0:0 | 0:0 | 0:0:P:0:0 |

Note: the index (the subject of the BOADICEA calculation) has the label 'T' in the Name column. genetic test type:result (type: 0=untested, S=mutation search, T=direct: result 0=untested, P=present, N=absent). pathology status, 0=unspecified, N=negative, P=positive

# Summary of Family and Age of Cancer Diagnoses

| **Name** | **Target** | **IndivID** | **FathID** | **MothID** | **Sex** | **MZtwin** | **Dead** | **Age** | **Yob** | **BC1** | **BC2** | **OC** | **PRO** | **PAN** | **Ashkn** |
| --- | --- | --- | --- | --- | --- | --- | --- | --- | --- | --- | --- | --- | --- | --- | --- |
| father | 0 | m21 | 0 | 0 | M | 0 | 0 | 72 | 1950 | 0 | 0 | 0 | 60 | 0 | 0 |
| NA | 0 | piLN | 0 | 0 | M | 0 | 0 | 0 | 0 | 0 | 0 | 0 | 0 | 0 | 0 |
| mother | 0 | f21 | 0 | 0 | F | 0 | 0 | 70 | 1952 | 50 | 0 | 0 | 0 | 0 | 0 |
| me | 1 | ch1 | m21 | f21 | F | 0 | 0 | 42 | 1980 | 0 | 0 | 0 | 0 | 0 | 0 |
| Sister | 0 | iKuE | m21 | f21 | F | 0 | 0 | 45 | 1977 | 0 | 0 | 0 | 0 | 0 | 0 |
| Brother | 0 | uZUG | m21 | f21 | M | 0 | 1 | 30 | 1977 | 0 | 0 | 0 | 0 | 0 | 0 |
| Son 1 | 0 | Zunu | piLN | ch1 | M | 0 | 0 | 17 | 2005 | 0 | 0 | 0 | 0 | 0 | 0 |
| Daughter 1 | 0 | sXNv | piLN | ch1 | F | 0 | 0 | 24 | 1998 | 0 | 0 | 0 | 0 | 0 | 0 |
| Son 2 | 0 | OQGB | piLN | ch1 | M | 0 | 0 | 12 | 2010 | 0 | 0 | 0 | 0 | 0 | 0 |

**Risk Category (NICE)**

Based on your risk assessment you are at **moderate risk**. Please refer to national screening guidelines.

# Recommendations for Managing Risk of Breast Cancer

The woman's lifetime risk from age 20 of having breast cancer is 18.9%. According to the NICE guidelines† the woman would be in the **moderate** risk category.

The woman's risk between ages 40 and 50 of having breast cancer is 2.4%. According to the NICE guidelines† the woman would be in the **population** risk category.

|  | **Near population risk** | **Moderate risk** | **High risk** |
| --- | --- | --- | --- |
| Lifetime risk from age 20 | Less than 17% | **17% or greater but less than 30%** | 30% or greater |
| Risk between ages 40 and 50 | **Less than 3%** | 3% or greater to 8% | Greater than 8% |


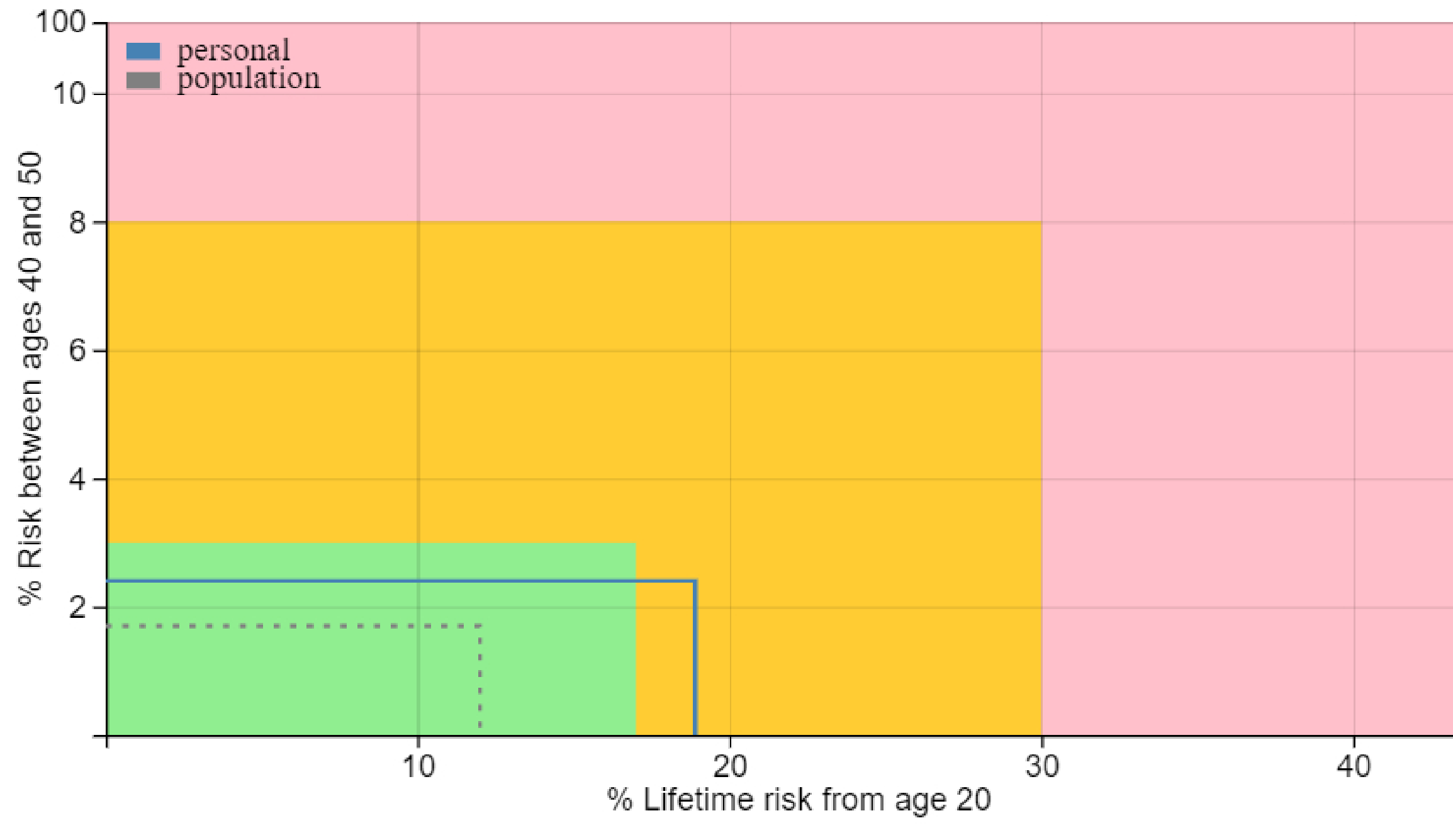


# Recommendations for Risk of Carrying a Pathogenic Mutation

The woman's probability of having a BRCA1 or BRCA2 mutation is 0.6%. The NICE guidelines† recommend offering genetic testing to people with a 10% (or bigger) likelihood of carrying a BRCA1/BRCA2 mutation.

# Breast Cancer Risk Absolute Risk of Breast Cancer from Current Age

The woman's risk of developing **breast cancer over the next 5 years is 1.1%**. In other words, about 11 out of 1000 women with these risk factors will develop cancer over the next 5 year period.

The woman's risk of developing **breast cancer over the next 10 years is 3.1%**. In other words, about 31 out of 1000 women with these risk factors will develop cancer over the next 10 year period.

The woman's risk of developing **breast cancer between 42 and 80 is 17.8%**. In other words, about 178 out of 1000 women with these risk factors will develop cancer by the age of 80.

| **Patient Age (years)** | **Breast Cancer Risk (%)** | **Population Risk (%)** |
| --- | --- | --- |
| 43 | 0.2 | 0.1 |
| 44 | 0.4 | 0.3 |
| 45 | 0.6 | 0.4 |
| 46 | 0.8 | 0.6 |
| 47 | 1.1 | 0.8 |
| 50 | 2.1 | 1.5 |
| 52 | 3.1 | 2 |
| 55 | 4.6 | 2.8 |
| 60 | 7.2 | 4.2 |
| 65 | 10 | 5.9 |
| 70 | 12.8 | 7.7 |
| 75 | 15.4 | 9.5 |
| 80 | 17.8 | 11.3 |

Population values are the risk in a random equivalent person in the population without any information on risk of genetic factors (i.e. based on population incidences only).

# Personal Risk of Developing Breast Cancer Compared to the Population

The woman's risk of developing **breast cancer by the age of 80 is 17.8%**, compared to the **average population risk of 11.3%**. In other words, about 178 out of 1000 women with these risk factors will develop breast cancer by the age of 80, compared to an average woman where 113 in 1000 will develop breast cancer.


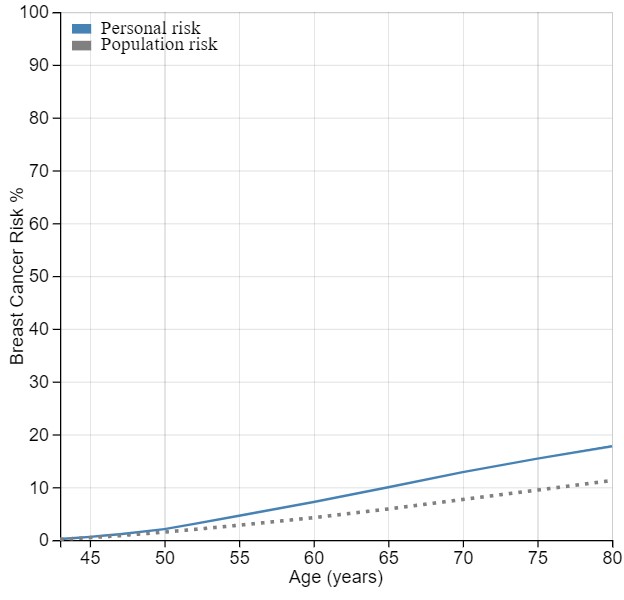


# Breast Cancer 10 Year Risk

The woman's risk of developing **breast cancer over the next 10 years is 3.1%**. In other words, about 31 out of 1000 women with these risk factors will develop cancer over the next 10 year period.


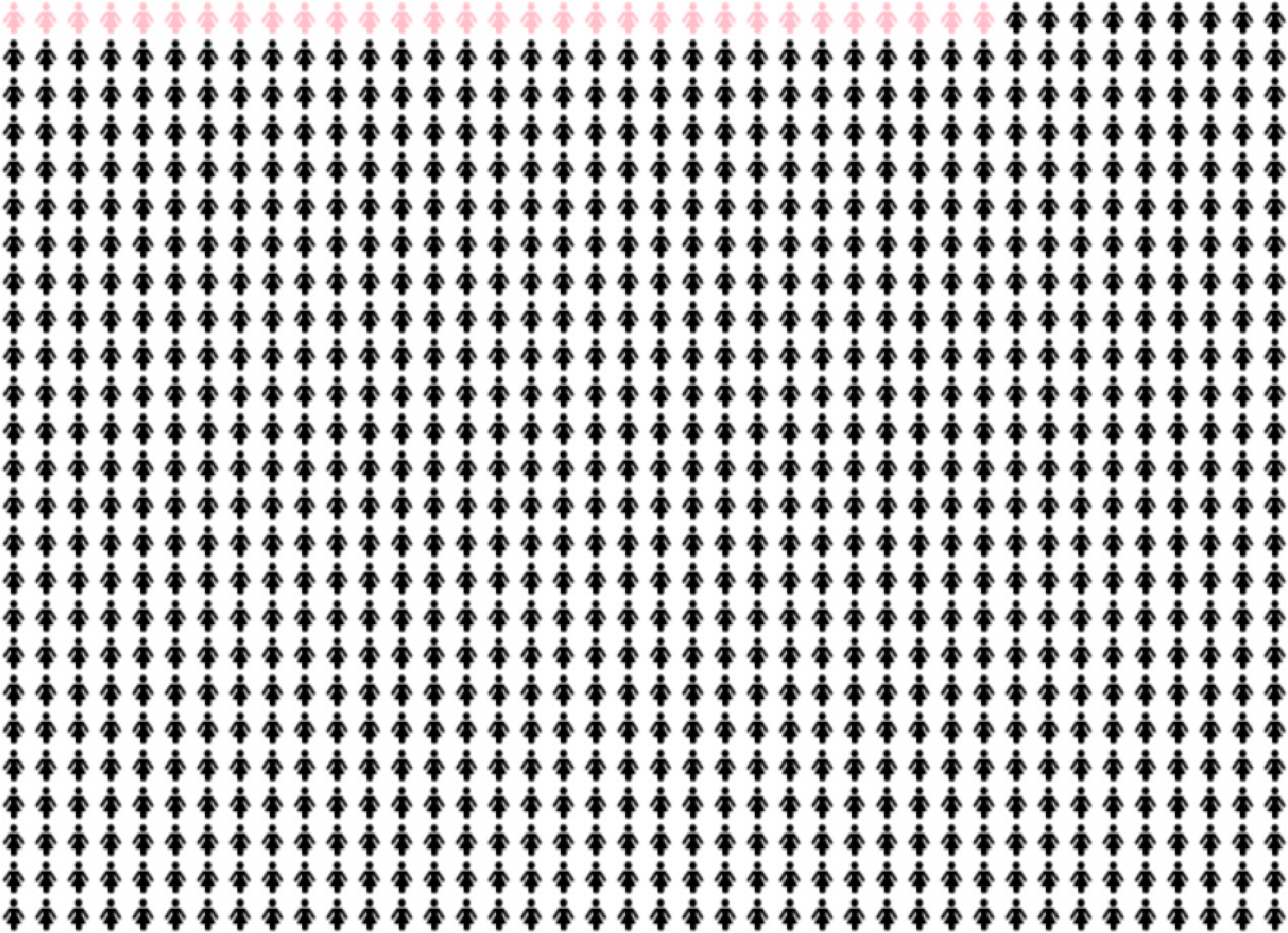


# Ovarian Cancer Risk Absolute Risk of Ovarian Cancer from Current Age

The woman's risk of developing **ovarian cancer over the next 5 years is 0%**. In other words, about 0 out of 1000 women with these risk factors will develop cancer over the next 5 year period.

The woman's risk of developing **ovarian cancer over the next 10 years is 0.1%**. In other words, about 1 out of 1000 women with these risk factors will develop cancer over the next 10 year period.

The woman's risk of developing **ovarian cancer between 42 and 80 is 1%**. In other words, about 10 out of 1000 women with these risk factors will develop cancer by the age of 80.

| **Patient Age (years)** | **Ovarian Cancer Risk (%)** | **Population Risk (%)** |
| --- | --- | --- |
| 43 | 0 | 0 |
| 44 | 0 | 0 |
| 45 | 0 | 0 |
| 46 | 0 | 0.1 |
| 47 | 0 | 0.1 |
| 50 | 0.1 | 0.1 |
| 52 | 0.1 | 0.2 |
| 55 | 0.2 | 0.3 |
| 60 | 0.3 | 0.5 |
| 65 | 0.4 | 0.7 |
| 70 | 0.6 | 1 |
| 75 | 0.8 | 1.3 |
| 80 | 1 | 1.7 |

Population values are the risk in a random equivalent person in the population without any information on risk of genetic factors (i.e. based on population incidences only).

# Personal Risk of Developing Ovarian Cancer Compared to the Population

The woman's risk of developing **ovarian cancer by the age of 80 is 1%**, compared to the **average population risk of 1.7%**. In other words, about 10 out of 1000 women with these risk factors will develop ovarian cancer by the age of 80, compared to an average woman where 17 in 1000 will develop ovarian cancer.


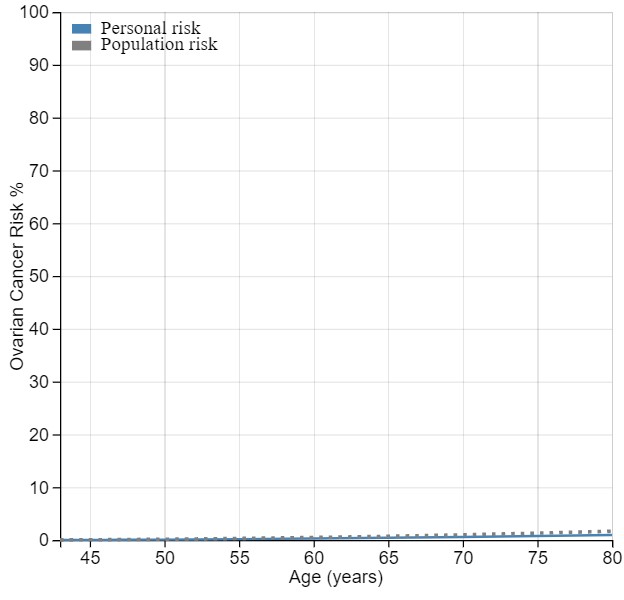


# Ovarian Cancer 10 Year Risk

The woman's risk of developing **ovarian cancer over the next 10 years is 0.1%**. In other words, about 1 out of 1000 women with these risk factors will develop cancer over the next 10 year period.


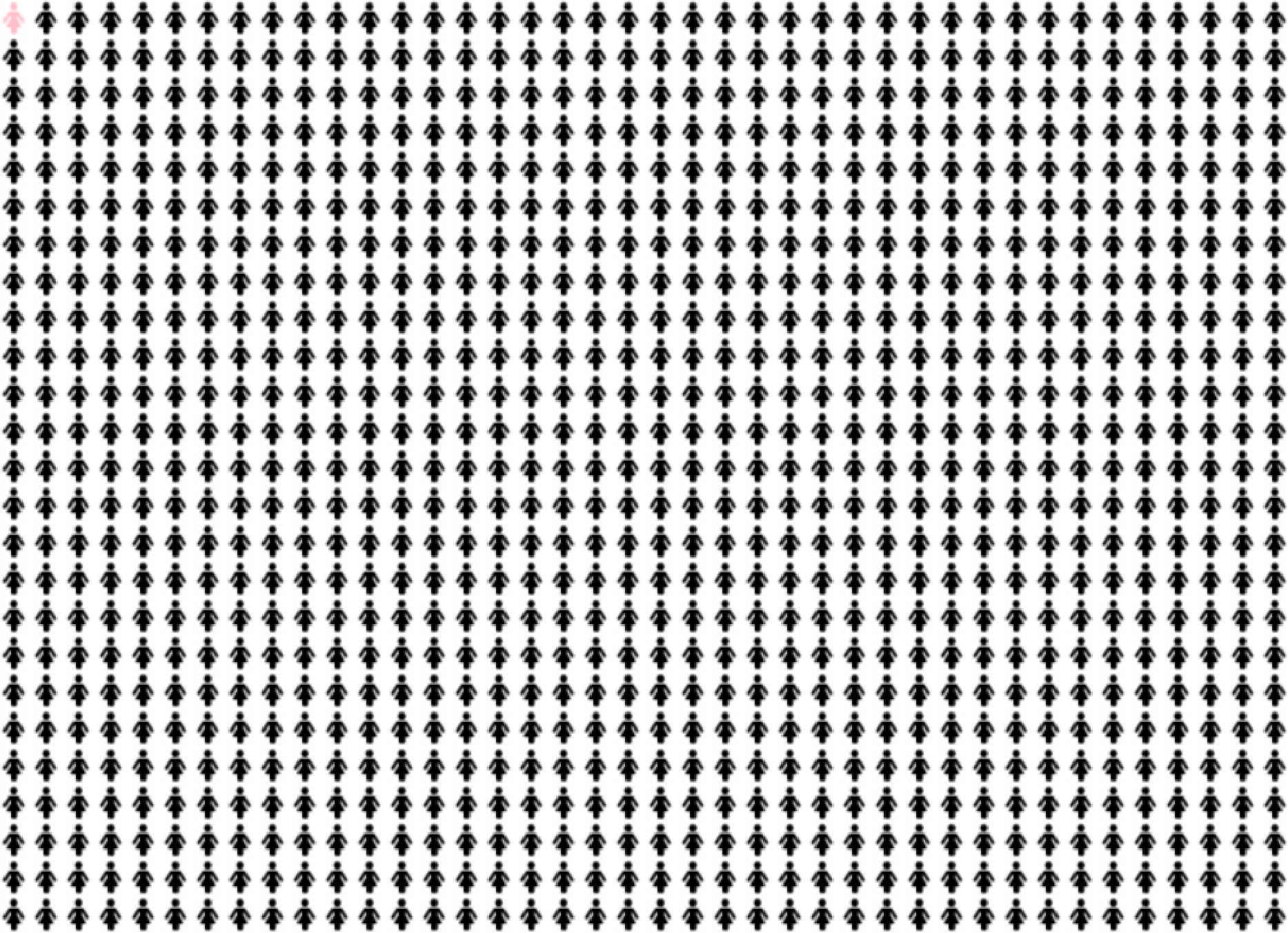


# Mutation Carrier Probability

From the breast cancer model, based on the woman's information, the mutation carrier probability for a pathogenic variant in:

- BRCA1 is 0.08%
- BRCA2 is 0.50%
- BRCA1 or BRCA2 is 0.58%
- PALB2 is 0.07%
- CHEK2 is 1.12%
- ATM is 0.51%
- BARD1 is 0.12%

From the ovarian cancer model, based on the woman's information, the mutation carrier probability for a pathogenic variant in:

- RAD51D is 0.09%
- RAD51C is 0.10%
- BRIP1 is 0.14%

This results in the carrier probability for a pathogenic variant in:

- any of the genes; BRCA1, BRCA2, PALB2, CHEK2, ATM, BARD1, RAD51D, RAD51C or BRIP1 genes is 2.73%
- none of the genes; BRCA1, BRCA2, PALB2, CHEK2, ATM, BARD1, RAD51D, RAD51C or BRIP1 genes is 97.27%

# Extra Information

For greater accuracy in the model, more information on the following people could have been included:

- year of birth and age at last follow up must be specified in order for unnamed to be included in a calculation * Incomplete data record in the pedigree: family member "mother" has an unspecified ER status, but another pathology parameter (PR, HER2, CK14 or CK5/6) has been specified. Please note the following rules for breast cancer pathology data: (1) if an individual's ER status is unspecified, no pathology information for that individual will be taken into account in the calculation; (2) if a breast cancer is ER positive, no other pathology information for that individual will be taken into account in the calculation; (3) if a breast cancer is ER negative, information on PR and HER2 for that individual will only be taken into account in the calculation if both PR and HER2 are specified; and (4) an individual's CK14 and CK5/6 status will only be taken into account in the calculation if both CK14 and CK5/6 are specified and the breast cancer is triple negative (ER negative, PR negative and HER2 negative). As a result, this individual's pathology information will not be taken into account in this case.
- Incomplete data record in the pedigree: family member "mother" has a breast cancer pathology where PR status isspecified but HER2 status is unspecified (or vice versa). Please note the following rules for breast cancer pathology data: (1) if an individual's ER status is unspecified, no pathology information for that individual will be taken into account in the calculation; (2) if a breast cancer is ER positive, no other pathology information for that individual will be taken into account in the calculation; (3) if a breast cancer is ER negative, information on PR and HER2 for that individual will only be taken into account in the calculation if both PR and HER2 are specified; and (4) an individual's CK14 and CK5/6 status will only be taken into account in the calculation if both CK14 and CK5/6 are specified and the breast cancer is triple negative (ER negative, PR negative and HER2 negative). As a result, PR and HER2 status will not be taken into account in this case.
