# Supplementary material for: Improving the communication of multifactorial cancer risk assessment results for different audiences: a co-design process
Source: J Community Genet. 2024 Sep 25;15(5):499–515. doi: 10.1007/s12687-024-00729-4 (PMC11549070; doi:10.1007/s12687-024-00729-4)
Supplement: Supplementary file 2 — Supplementary file2 (DOCX 449 KB) [file 12687_2024_729_MOESM2_ESM.docx]

**New CanRisk Report**

**Please ignore risk numbers and graph/icon information as these have been taken from different sources so do not match.**

**Introduction:**

This report presents the results of your CanRisk assessment and it is split into **three sections**:

As you have already had a diagnosis of breast cancer, the risks presented in this report are for contralateral breast cancer, that is, of the other (unaffected) breast. When your risks are compared to the risk of the population, this refers to the risk of average women in the UK who have already had breast cancer.

**Section one** presents:

- information about your risk of developing breast cancer in the next 5 years, 10 years and between now and the age of 80. These risk results are based on the range of information you provided about yourself and your family.
- information about your risk of developing ovarian cancer in the next 5 years, 10 years and between now and the age of 80. These risk results are based on the range of information you provided about yourself and your family.

**Sections two and three present more specific information that might be more relevant to your healthcare professional.**

**Section two** presents:

- your risk of developing breast cancer throughout your lifetime, that is, between the ages of 20 and 80 and specifically between the ages of 40 and 50.
- your risk of developing breast cancer between now and the age of 80.
- your risk of developing ovarian cancer between now and the age of 80.
- your risk of carrying a genetic pathogenic variant (sometimes called a genetic mutation) that is relevant to your risk of developing breast and ovarian cancer in the future.

**Section three** presents:

- a visual representation of your family called a pedigree
- a summary of cancer diagnoses in your family based on the information you provided
- a summary of the other information included in the model used to calculate your breast cancer risks.
- your breast cancer polygenic risk score.
- a summary of the other information included in the model used to calculate your ovarian cancer risks.
- your ovarian cancer polygenic risk scores.

This report does not include healthcare recommendations to manage or reduce your risk of developing breast [and ovarian] cancer.

**SECTION 1:**

**Your risk of developing breast cancer**


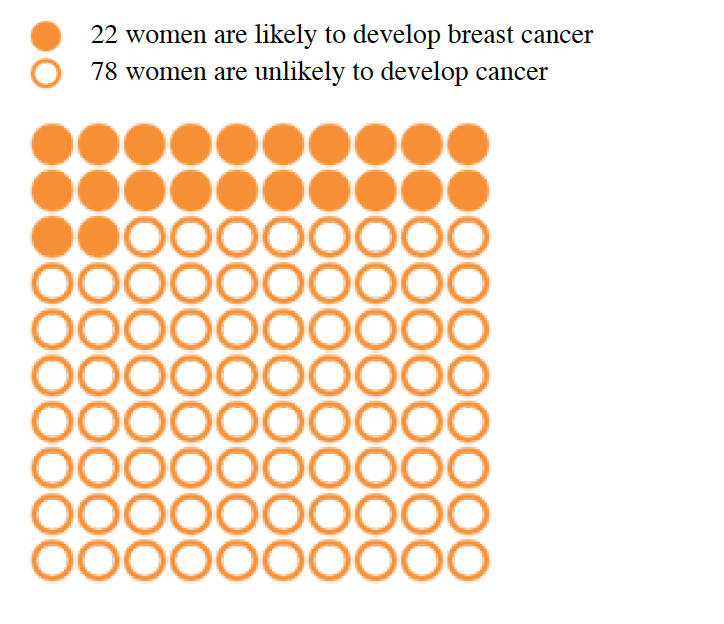
Your risk of developing **breast cancer over the next 5 years is 1.1%**. In other words, about 1 out of 100 women with the same risk factors as you will develop cancer over the next 5-year period. The image below might help you visualise this information.


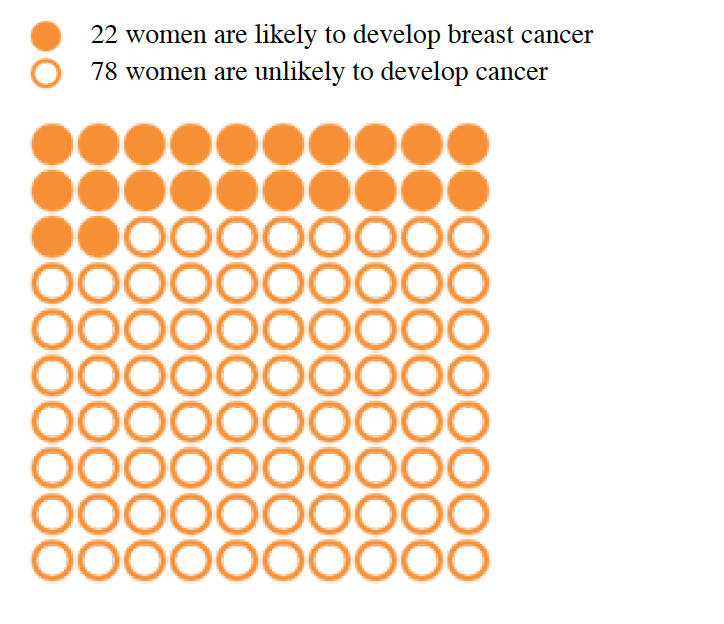
Your risk of developing **breast cancer over the next 10 years is 3.1%**. In other words, about 3 out of 100 women with the same risk factors as you will develop cancer over the next 10-year period. The image below might help you visualise this information.

Your risk of developing **breast cancer between now and when you are 80 years old is 17.8%**. In other words, about 18 out of 100 women with the same risk factors as you will develop cancer by the age of 80. The icon below might help you visualise this information.


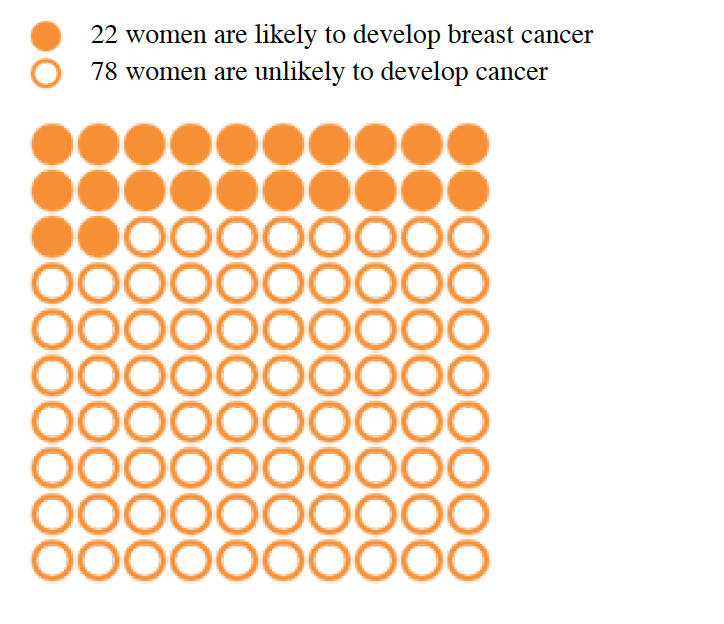


**Your breast cancer risks compared to the rest of the population:**

The following graph represents your remaining lifetime risk of developing breast cancer compared to the population. In other words, the graph shows your personal risk of developing breast cancer compared to the average risk of women in the UK (population risk)^[[1]](#footnote-1)^. The red dots in the graph mark the risk scores already presented for you in the next 5 years, 10 years and by age 80.

**
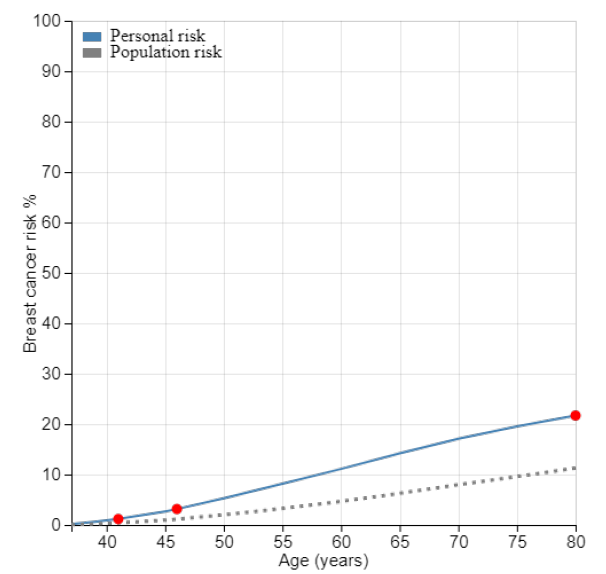
**

**Your breast cancer risk category (NICE):**

Based on your risk assessment and according to the NICE guidelines^[[2]](#footnote-2)^ you are at **moderate risk of developing breast cancer**. This means that based on the information you provided, you are at an increased risk of developing breast cancer compared to that of the average population in the UK. Your risk category may change if your risk factors change.

[Based on your risk assessment and according to the NICE guidelines^2^ you are at **population risk of developing breast cancer**. This means that based on the information you provided, you are at a similar or lower risk of developing breast cancer compared to that of the average population in the UK. Your risk may change if your risk factors change.]

**Your risk of developing ovarian cancer**

Your risk of developing **ovarian cancer over the next 5 years is 1.1%**. In other words, about 1 out of 100 women with the same risk factors as you will develop cancer over the next 5-year period. The image below might help you visualise this information.


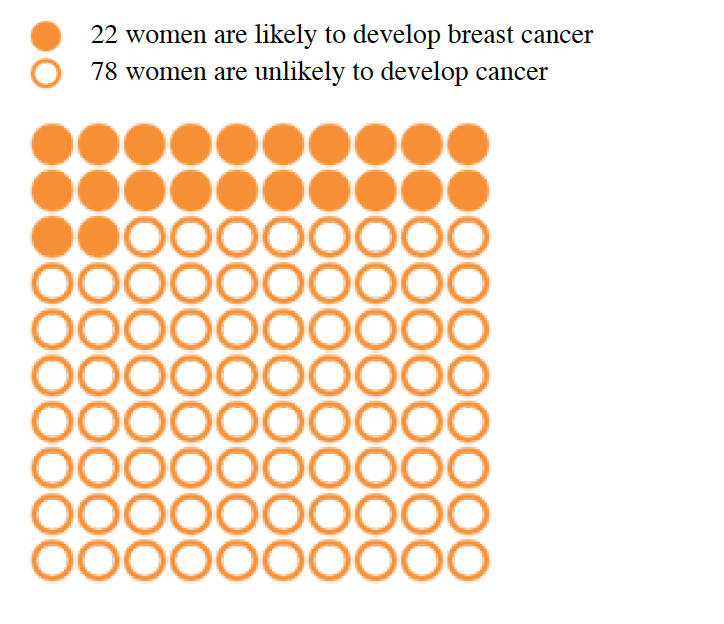


Your risk of developing **ovarian cancer over the next 10 years is 3.1%**. In other words, about 3 out of 100 women with the same risk factors as you will develop cancer over the next 10-year period. The image below might help you visualise this information.


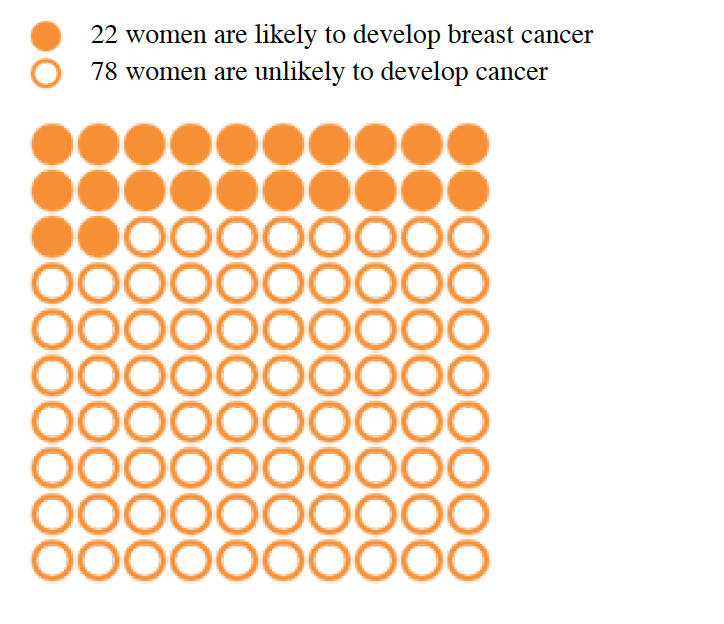


Your risk of developing **ovarian cancer between now and when you are 80 years old is 17.8%**. In other words, about 18 out of 100 women with the same risk factors as you will develop cancer by the age of 80. The icon below might help you visualise this information.


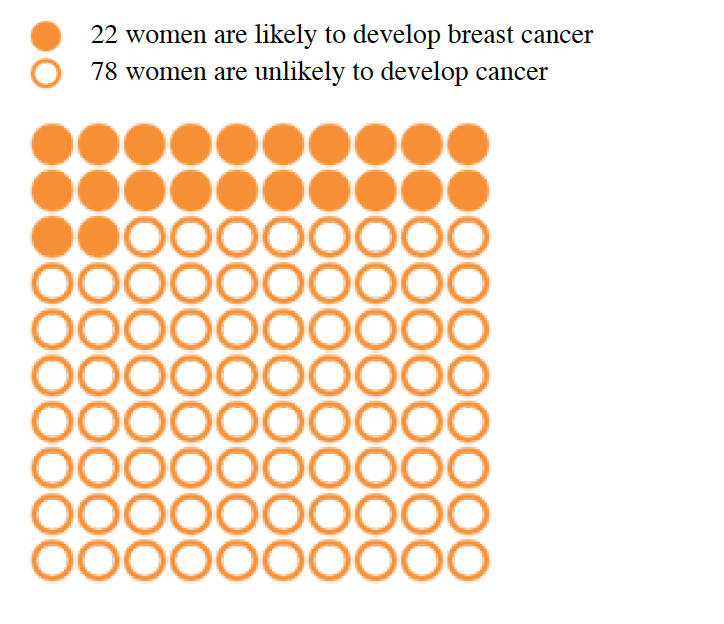


**Your ovarian cancer risks compared to the rest of the population:**

The following graph represents your remaining lifetime risk of developing ovarian cancer compared to the population risk. In other words, the graph shows your personal risk of developing ovarian cancer compared to the average risk of women in the UK (population risk)^[[3]](#footnote-3)^. The red dots in the graph mark the risk scores already presented for you in the next 5 years, 10 years and by age 80.

**
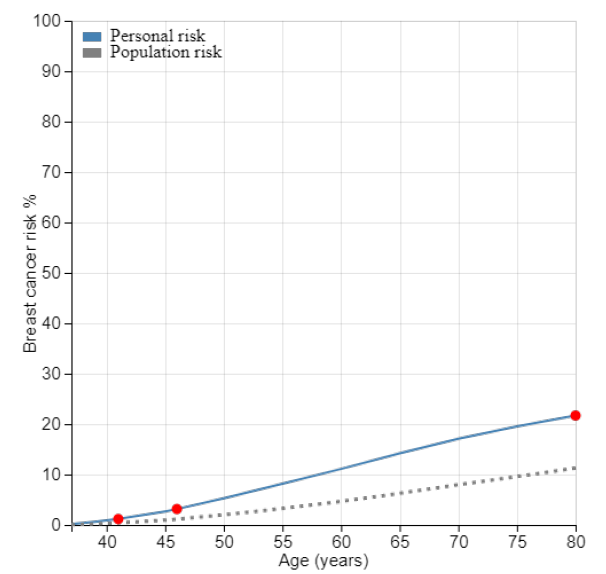
**

**SECTION 2:**

**Further information about your risk of developing breast cancer**

Your risk category is based on your lifetime risk of developing breast cancer, that is between the ages of 20 and 80 years and/or your risk of developing breast cancer between the ages of 40 and 50 years, whichever is higher.

Your lifetime risk of developing breast cancer is 18.9%. According to the NICE guidelines this puts you in the moderate risk category.

Your risk of developing breast cancer between ages of 40 and 50 years is 2.4%. According to the NICE guidelines this puts you in the population risk category.

The table below tells you the ranges for the three risk categories: near population risk, moderate risk and high risk.

|  | **Near population risk** | **Moderate risk** | **High risk** |
| --- | --- | --- | --- |
| Lifetime risk from age 20 | Less than 17% | **17% or greater but less than 30%** | 30% or greater |
| Risk between ages 40 and 50 | **Less than 3%** | 3% or greater to 8% | Greater than 8% |

The graph below shows the different risk categories as well as your personal lifetime risk and your personal risk between the ages of 40 and 50 of developing breast cancer compared to that of the population.


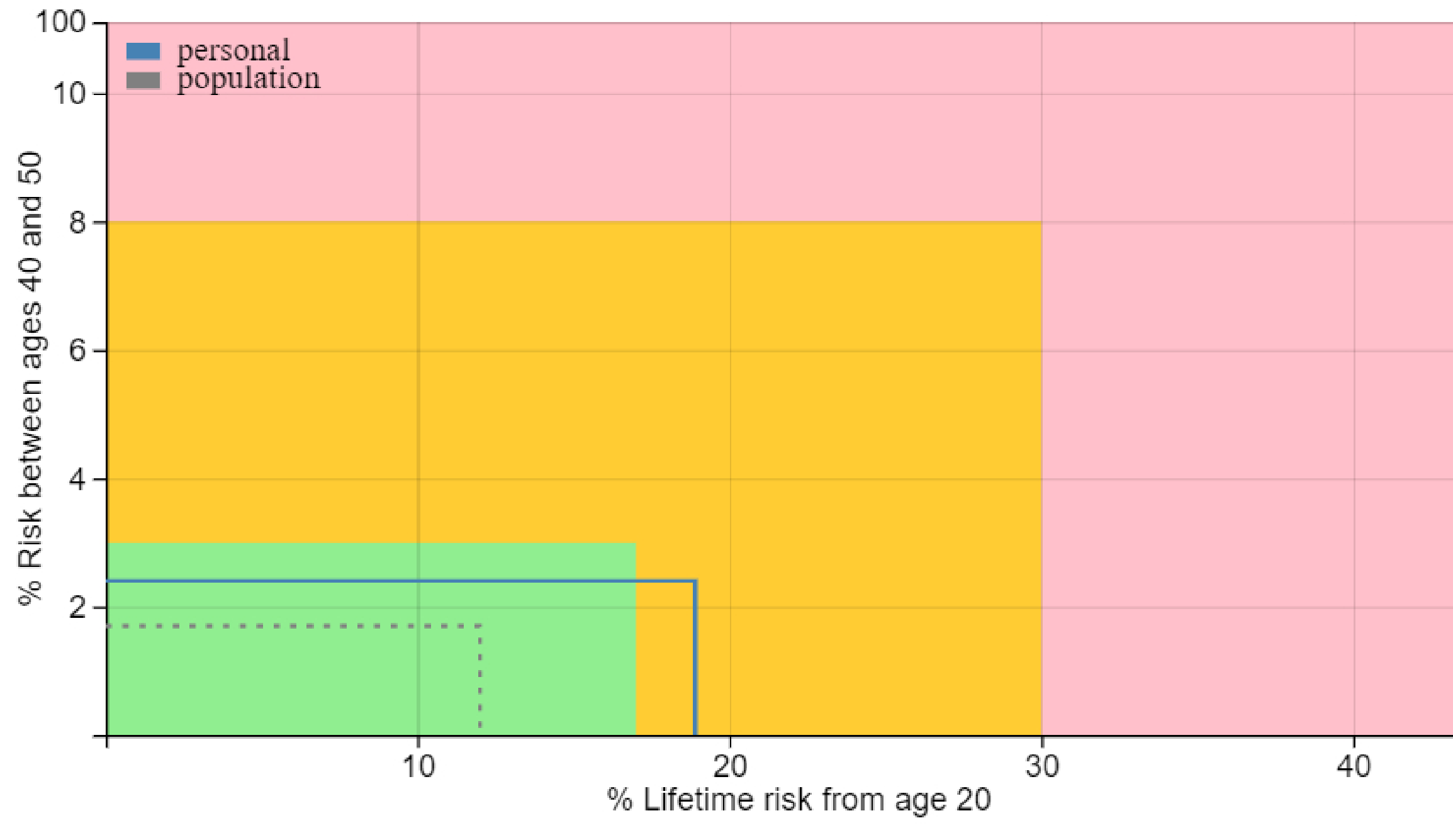


# Your remaining lifetime risk of developing breast cancer compared to the population:

Your risk of developing breast cancer between now and the age of 80 years is 17.8%, compared to the average population risk of 11.3%. In other words, about 18 out of 100 women with the same risk factors as you will develop breast cancer by the age of 80 years, compared to the population average of about 11 in 100 women in the UK. The table below shows how your risk changes over time.

| **Patient Age (years)** | **Breast Cancer Risk (%)** | **Population Risk (%)** |
| --- | --- | --- |
| 43 | 0.2 | 0.1 |
| 44 | 0.4 | 0.3 |
| 45 | 0.6 | 0.4 |
| 46 | 0.8 | 0.6 |
| 47 | 1.1 | 0.8 |
| 50 | 2.1 | 1.5 |
| 52 | 3.1 | 2 |
| 55 | 4.6 | 2.8 |
| 60 | 7.2 | 4.2 |
| 65 | 10 | 5.9 |
| 70 | 12.8 | 7.7 |
| 75 | 15.4 | 9.5 |
| 80 | 17.8 | 11.3 |

# Your remaining lifetime risk of developing ovarian cancer compared to the population:

Your risk of developing ovarian cancer between now and the age of 80 years is 17.8%, compared to the average population risk of 11.3%. In other words, about 18 out of 100 women with your risk factors will develop ovarian cancer by the age of 80 years, compared to the population average of about 11 in 100 women in the UK. The table below shows how your risk changes over time.

| **Patient Age (years)** | **Ovarian Cancer Risk (%)** | **Population Risk (%)** |
| --- | --- | --- |
| 43 | 0.2 | 0.1 |
| 44 | 0.4 | 0.3 |
| 45 | 0.6 | 0.4 |
| 46 | 0.8 | 0.6 |
| 47 | 1.1 | 0.8 |
| 50 | 2.1 | 1.5 |
| 52 | 3.1 | 2 |
| 55 | 4.6 | 2.8 |
| 60 | 7.2 | 4.2 |
| 65 | 10 | 5.9 |
| 70 | 12.8 | 7.7 |
| 75 | 15.4 | 9.5 |
| 80 | 17.8 | 11.3 |

# Your risk of carrying a genetic pathogenic variant:

# A genetic pathogenic variant is an alteration in a gene that is responsible for, or can contribute to, the development of disease. Below you will find the probability (or your chance) of carrying genetic a pathogenic variant in a gene that has been associated with an increased risk of developing breast and or ovarian cancer.

Your variation carrier probability for a genetic pathogenic variant in:

- BRCA1 is 0.08%
- BRCA2 is 0.50%
- BRCA1 or BRCA2 is 0.58%
- PALB2 is 0.07%
- CHEK2 is 1.12%
- ATM is 0.51%
- BARD1 is 0.12%
- RAD51D is 0.09%
- RAD51C is 0.10%
- BRIP1 is 0.14%

Your probability of carrying a pathogenic variant in:

Any of the genes (BRCA1, BRCA2, PALB2, CHEK2, ATM, BARD1, RAD51D, RAD51C or BRIP1) is 2.73%

None of the genes (BRCA1, BRCA2, PALB2, CHEK2, ATM, BARD1, RAD51D, RAD51C or BRIP1) is 97.23

**SECTION 3:**

**Your family pedigree:**

The image below is a visual representation of your family.


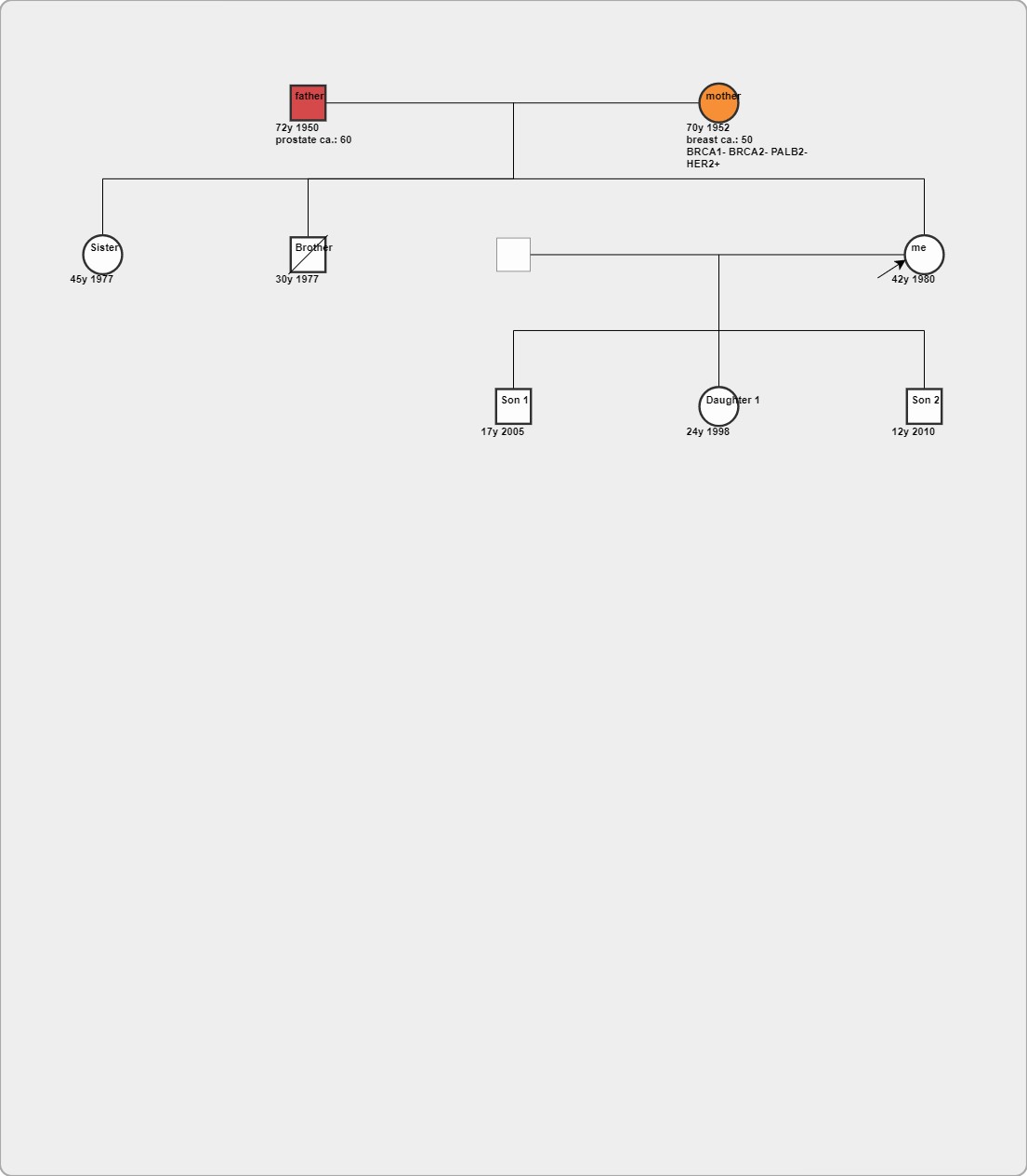


# Summary of genetic tests

The table below presents the results of genetic tests in your family.

|  |  | **Genetic Tests†** | |  | | |  |  |  |  | **Pathology** |
| --- | --- | --- | --- | --- | --- | --- | --- | --- | --- | --- | --- |
| **Name** |  | **BRCA1** | **BRCA2** | **PALB2** | **ATM** | **CHEK2** | **BARD1** | **RAD51D** | **RAD51C** | **BRIP1** | **ER:PR:HER2:CK14:CK56‡** |
| mother |  | S:N | S:N | S:N | 0:0 | 0:0 | 0:0 | 0:0 | 0:0 | 0:0 | 0:0:P:0:0 |

Note: the index (the subject of the CanRisk calculation) has the label 'T' in the Name column. Genetic test results for each gene shown in the following format: “type of genetic testing: result of the genetic test” (type can take the following values: 0=untested, S=mutation search, T=direct; result can take the following values: 0=untested, P=present, N=absent).

Breast cancer pathology status results in the following format **ER:PR:HER2:CK14:CK5**, with the following values for each marker: 0=unspecified, N=negative, P=positive

**The breast cancer model**

The information in the table below was used to calculate your risk of breast cancer.

| **Risk Factor** | | **Value** |
| --- | --- | --- |
| Age at First Occurrence of Menstruation | | 11 |
| Number of Children | | >2 |
| Age of First Live Birth | | <20 |
| Oral Contraception Usage | | former |
| Hormone Replacement Therapy | | never/former |
| Body Mass Index | | >=30 |
| Alcohol Intake (grams/day) | | 25-<35 |
| Age of Menopause | | - |
| Mammographic Density | | - |
| Height (cm) | | 167.64000000000001 |
| **Gene** | **Mutation frequency** | **Mutation sensitivity** |
| BRCA1 | 0.0006394 | 0.89 |
| BRCA2 | 0.00102 | 0.96 |
| PALB2 | 0.00064 | 0.92 |
| ATM | 0.0018 | 0.94 |
| CHEK2 | 0.00373 | 0.98 |
| BARD1 | 0.00043 | 0.89 |
| RAD51C | 0.00035 | 0.78 |
| RAD51D | 0.00035 | 0.86 |

Note, the following parameter settings were used:

Pathogenic variant frequencies: UK

Cancer incidence rates: UK

The model has been developed using data from European ancestry populations.

**Breast cancer polygenic scores:**

Polygenic scores add together small genetic changes in someone’s genetic code (DNA) to help estimate their likelihood of getting a disease, such as breast cancer.

**6.3%** of people in the population have a **lower** polygenic score than you, and **93.7%** of people in the population have a **higher** polygenic score than you. The graph below might help you visualise this information.

#

Note: breast cancer polygenic scores are calculated using data from European ancestry populations. The parameters used for this calculation are alpha: 0.441 and z-score: -1.52

# The ovarian cancer model

The information in the table below was used to calculate your risk of ovarian cancer.

**
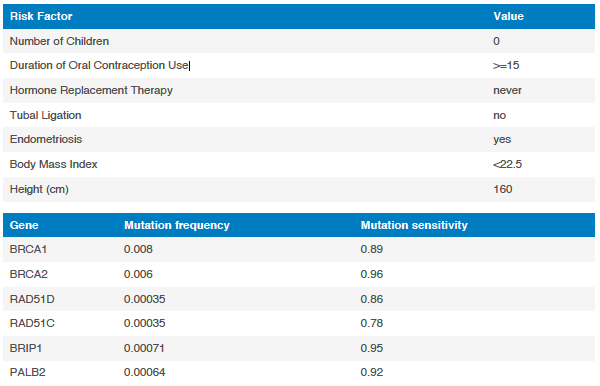
**

Note, the following parameter settings were used:

Pathogenic variant frequencies UK

Cancer incidence rates: UK

The model has been developed using data from European ancestry populations.

**Ovarian cancer polygenic scores:**

Polygenic scores add together small genetic changes in someone’s genetic code (DNA) to help estimate their likelihood of getting a disease, such as ovarian cancer.

**8.3%** of people in the population have a **lower** polygenic score than you, and **91.7%** of people in the population have a **higher** polygenic score than you. The graph below might help you visualise this information.

Note: ovarian cancer polygenic scores are calculated using data from European ancestry populations. The parameters used in this calculation are alpha: 0.219 and z-score: -1.38

**Summary of cancer diagnoses in your family**

The table below summarises information about cancer diagnoses in your family and how old your family members were when they were diagnosed.

| **Name** | **Target** | **IndivID** | **FathID** | **MothID** | **Sex** | **MZtwin** | **Dead** | **Age** | **Yob** | **BC1** | **BC2** | **OC** | **PRO** | **PAN** | **Ashkn** |
| --- | --- | --- | --- | --- | --- | --- | --- | --- | --- | --- | --- | --- | --- | --- | --- |
| father | 0 | m21 | 0 | 0 | M | 0 | 0 | 72 | 1950 | 0 | 0 | 0 | 60 | 0 | 0 |
| NA | 0 | piLN | 0 | 0 | M | 0 | 0 | 0 | 0 | 0 | 0 | 0 | 0 | 0 | 0 |
| mother | 0 | f21 | 0 | 0 | F | 0 | 0 | 70 | 1952 | 50 | 0 | 0 | 0 | 0 | 0 |
| me | 1 | ch1 | m21 | f21 | F | 0 | 0 | 42 | 1980 | 0 | 0 | 0 | 0 | 0 | 0 |
| Sister | 0 | iKuE | m21 | f21 | F | 0 | 0 | 45 | 1977 | 0 | 0 | 0 | 0 | 0 | 0 |
| Brother | 0 | uZUG | m21 | f21 | M | 0 | 1 | 30 | 1977 | 0 | 0 | 0 | 0 | 0 | 0 |
| Son 1 | 0 | Zunu | piLN | ch1 | M | 0 | 0 | 17 | 2005 | 0 | 0 | 0 | 0 | 0 | 0 |
| Daughter 1 | 0 | sXNv | piLN | ch1 | F | 0 | 0 | 24 | 1998 | 0 | 0 | 0 | 0 | 0 | 0 |
| Son 2 | 0 | OQGB | piLN | ch1 | M | 0 | 0 | 12 | 2010 | 0 | 0 | 0 | 0 | 0 | 0 |

Note: ‘Target’ is the woman undergoing the risk assessment; ‘IndivID’ is the unique ID of the family member; ‘FathID’ is the unique ID of the father; ‘MothID’ is the unique ID of the mother; ‘Sex’ is the sex of the person; ‘MZtwin’ means identical twins; ‘Dead’ is whether the person is dead; ‘Yob’ is the person’s year of birth; ‘BC1’ is the age at first breast cancer diagnosis; ‘BC2’ is the age at second (contralateral) breast cancer diagnosis; ‘OC’ is age at ovarian cancer diagnosis; ‘PRO’ is age at prostate cancer diagnosis; ‘PAN’ is age at pancreatic cancer diagnosis and ‘Ashkn’ is Ashkenazi status.

# Extra information

For greater accuracy in the model, more information on the following people could have been included:

- year of birth and age at last follow up must be specified in order for unnamed to be included in a calculation * Incomplete data record in the pedigree: family member "mother" has an unspecified ER status, but another pathology parameter (PR, HER2, CK14 or CK5/6) has been specified. Please note the following rules for breast cancer pathology data: (1) if an individual's ER status is unspecified, no pathology information for that individual will be taken into account in the calculation; (2) if a breast cancer is ER positive, no other pathology information for that individual will be taken into account in the calculation; (3) if a breast cancer is ER negative, information on PR and HER2 for that individual will only be taken into account in the calculation if both PR and HER2 are specified; and (4) an individual's CK14 and CK5/6 status will only be taken into account in the calculation if both CK14 and CK5/6 are specified and the breast cancer is triple negative (ER negative, PR negative and HER2 negative). As a result, this individual's pathology information will not be taken into account in this case.
- Incomplete data record in the pedigree: family member "mother" has a breast cancer pathology where PR status is specified but HER2 status is unspecified (or vice versa). Please note the following rules for breast cancer pathology data: (1) if an individual's ER status is unspecified, no pathology information for that individual will be taken into account in the calculation; (2) if a breast cancer is ER positive, no other pathology information for that individual will be taken into account in the calculation; (3) if a breast cancer is ER negative, information on PR and HER2 for that individual will only be taken into account in the calculation if both PR and HER2 are specified; and (4) an individual's CK14 and CK5/6 status will only be taken into account in the calculation if both CK14 and CK5/6 are specified and the breast cancer is triple negative (ER negative, PR negative and HER2 negative). As a result, PR and HER2 status will not be taken into account in this case.

1. Note: (see comment on the right/hand side of this page specifying the content of this footnote for different groups) [↑](#footnote-ref-1)
2. NICE is the National Institute for Health and Care Excellence in the UK and it provides evidence-based recommendations developed by independent committees, including professionals and lay members, and consulted on by stakeholders. [↑](#footnote-ref-2)
3. Note: for women who have not had a breast cancer diagnosis, population values are the risk in a random equivalent person in the population without any information on risk of genetic factors (i.e. based on population incidences only). [↑](#footnote-ref-3)
