# Supplementary material for: Improving the communication of multifactorial cancer risk assessment results for different audiences: a co-design process
Source: J Community Genet. 2024 Sep 25;15(5):499–515. doi: 10.1007/s12687-024-00729-4 (PMC11549070; doi:10.1007/s12687-024-00729-4)
Supplement: Supplementary file 3 — Supplementary file3 (PDF 452 KB) [file 12687_2024_729_MOESM3_ESM.pdf]

CanRisk Report

This report presents the results of your CanRisk assessment and it has three sections.

Section one presents:

- information about your risk of developing breast cancer in the next 5 years, 10 years and between now and the age of 80. These risk results are based on the information you provided about yourself and your family.
- information about your risk of developing ovarian cancer in the next 5 years, 10 years and between now and the age of 80. These risk results are based on the information you provided about yourself and your family.

Sections two and three present more specific information that might be more relevant to your healthcare professional.

Section two presents:

- further information about your risk of developing breast cancer between the ages of 20 and 80 and between the ages of 40 and 50.
- further information about your risk of developing breast cancer between now and the age of 80.
- further information about your risk of developing ovarian cancer between now and the age of 80.
- your risk of carrying a genetic pathogenic variant (or genetic mutation) that is relevant to your risk of developing breast and ovarian cancer in the future.

Section three presents:

- a visual representation of your family (called a pedigree) based on the information you provided.
- a summary of cancer diagnoses in your family based on the information you provided.
- a summary of the other information included in the model used to calculate your breast cancer risks.
- your breast cancer polygenic score.
- a summary of the other information included in the model used to calculate your ovarian cancer risks.

This report does not include healthcare recommendations to manage or reduce your risk of developing breast and ovarian cancer. Your risk is calculated based on the information you have provided. Note that your risk may change if your risk factors change (more information on risk factors in section 3).

SECTION 1: Information relevant for you

Your Risk of Developing Breast Cancer

Your risk of developing **breast cancer over the next 5 years is 1.2%**. In other words, about 1 out of 100 women with these risk factors will develop cancer over the next 5 year period. The image below might help you visualise this information.

1 women are likely to develop breast cancer
99 women are unlikely to develop breast cancer

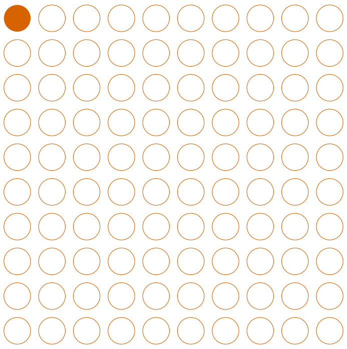

Your risk of developing **breast cancer over the next 10 years is 3.1%**. In other words, about 3 out of 100 women with these risk factors will develop cancer over the next 10 year period. The image below might help you visualise this information.

3 women are likely to develop breast cancer
97 women are unlikely to develop breast cancer

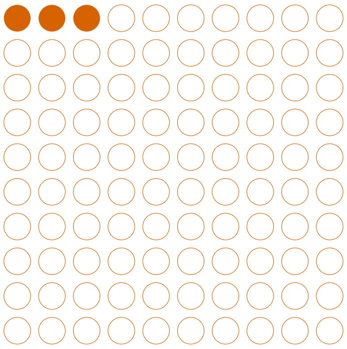

Your risk of developing **breast cancer between now and when you are 80 years old is 15.4%**. In other words, about 15 out of 100 women with these risk factors will develop cancer by the age of 80. The image below might help you visualise this information.

15 women are likely to develop breast cancer
85 women are unlikely to develop breast cancer

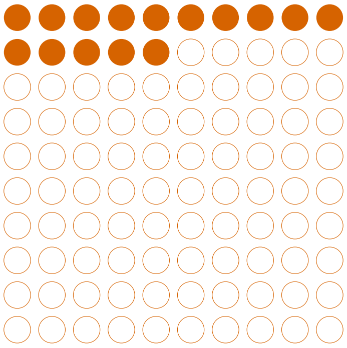

Your Breast Cancer Risks Compared to the Rest of the Population

The graph represents your risk of developing breast cancer between now and the age of 80 years compared to the population. In other words, the graph shows your personal risk of developing breast cancer compared to the average risk of women in the population. The red dots in the graph mark the risk scores already presented for you in the next 5 years, 10 years and by age 80.

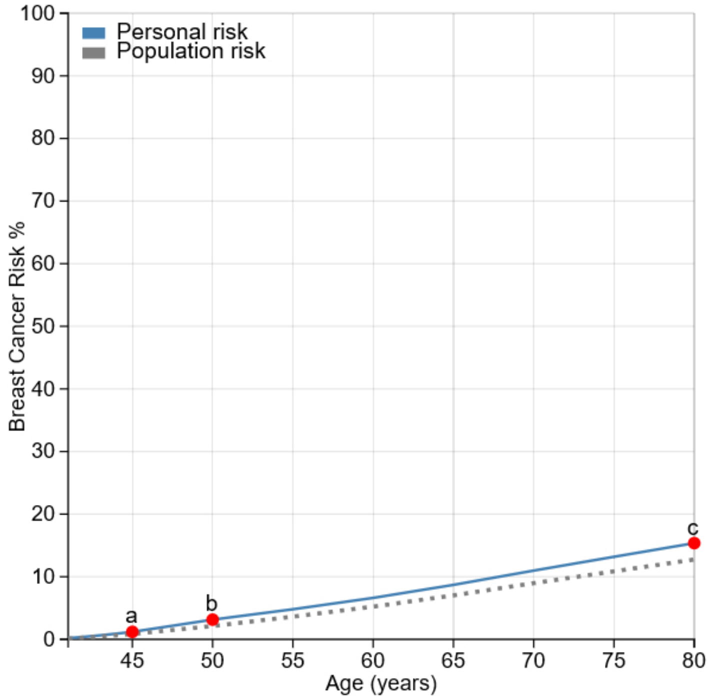

Graph key:

Label	Your breast cancer risk
a	Next 5 year risk is 1.2%
b	Next 10 year risk is 3.1%
c	Risk between now and the age of 80 is 15.4%

Note: population values are the risk in a random equivalent person in the population without any information on risk of genetic factors (i.e. based on population incidences only).

Below is an alternative way of visualising your risk of developing breast cancer between now and the age of 80 years compared to the average population risk.

Your risk 15.4%

- 15 women are likely to develop breast cancer
- 85 women are unlikely to develop breast cancer

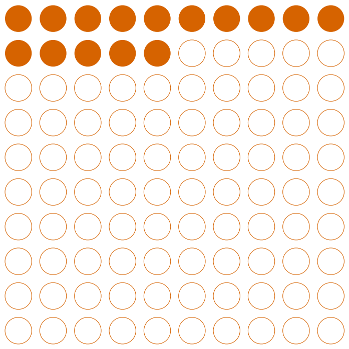

Population risk 12.8%

- 13 women are likely to develop breast cancer
- 87 women are unlikely to develop breast cancer

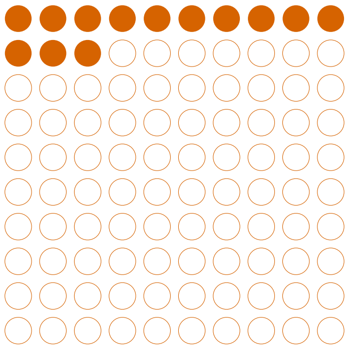

Your breast cancer risk category (NICE):

Based on your risk assessment and according to the NICE guidelines you are at **moderate risk of developing breast cancer**. This means that based on the information you provided, you are at an increased risk of developing breast cancer compared to that of the average population in the UK. Your risk category may change if your risk factors change.

More information about this in section 2.

Your Risk of Developing Ovarian Cancer

Your risk of developing **ovarian cancer over the next 5 years is 0.1%**. In other words, about 0 out of 100 women with these risk factors will develop cancer over the next 5 year period. The image below might help you visualise this information.

0 women are likely to develop ovarian cancer
100 women are unlikely to develop ovarian cancer

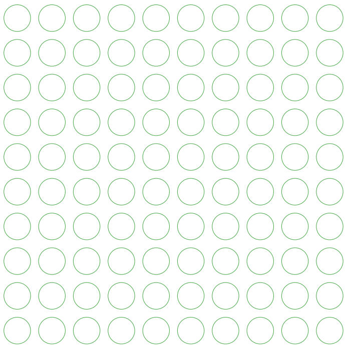

Your risk of developing **ovarian cancer over the next 10 years is 0.3%**. In other words, about 0 out of 100 women with these risk factors will develop cancer over the next 10 year period. The image below might help you visualise this information.

0 women are likely to develop ovarian cancer
100 women are unlikely to develop ovarian cancer

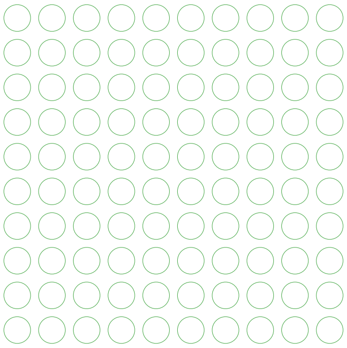

Your risk of developing **ovarian cancer between now and when you are 80 years old is 2.3%**. In other words, about 2 out of 100 women with these risk factors will develop cancer by the age of 80. The image below might help you visualise this information.

2 women are likely to develop ovarian cancer
98 women are unlikely to develop ovarian cancer

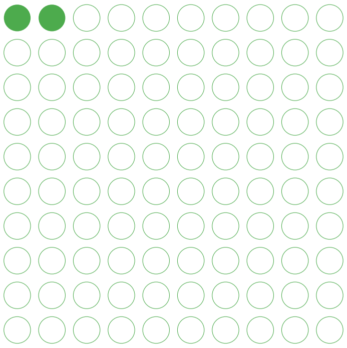

Your Ovarian Cancer Risks Compared to the Rest of the Population

The graph represents your risk of developing ovarian cancer between now and the age of 80 years compared to the population. In other words, the graph shows your personal risk of developing ovarian cancer compared to the average risk of women in the population. The red dots in the graph mark the risk scores already presented for you in the next 5 years, 10 years and by age 80.

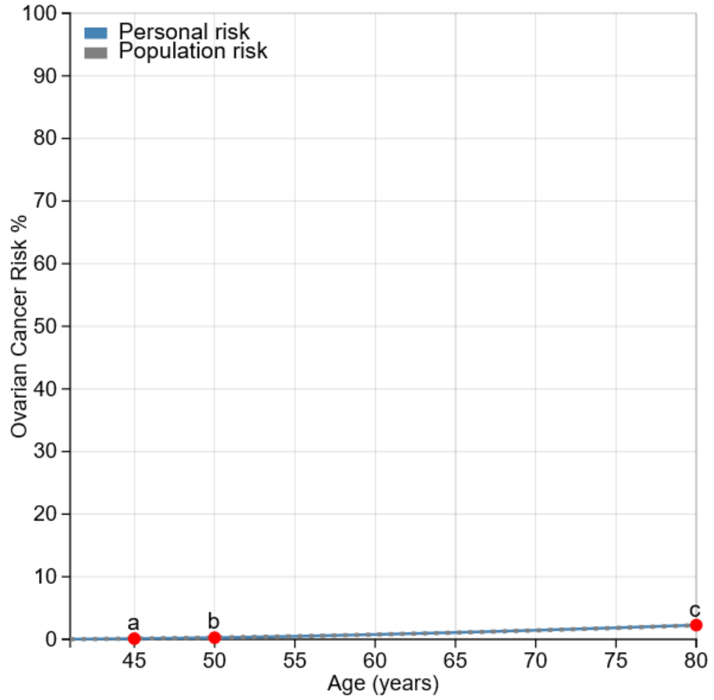

Graph key:

Label	Your breast cancer risk
a	Next 5 year risk is 1.2%
b	Next 10 year risk is 3.1%
c	Risk between now and the age of 80 is 15.4%

Note: population values are the risk in a random equivalent person in the population without any information on risk of genetic factors (i.e. based on population incidences only).

Below is an alternative way of visualising your risk of developing ovarian cancer between now and the age of 80 years compared to the average population risk.

Your risk 2.3%

- 2 women are likely to develop ovarian cancer
- 98 women are unlikely to develop ovarian cancer

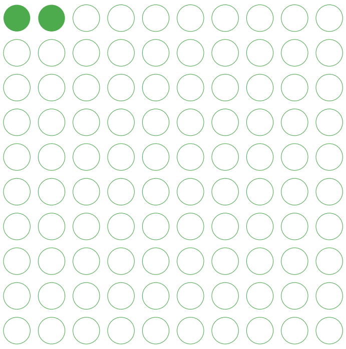

Population risk 2.2%

- 2 women are likely to develop ovarian cancer
- 98 women are unlikely to develop ovarian cancer

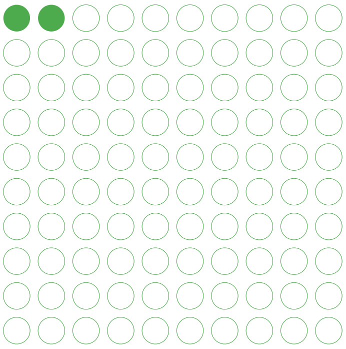

SECTION 2: Information more relevant for healthcare professionals

Further Information About Your Risk of Developing Breast Cancer

Your risk category is based on your risk of developing breast cancer between the ages of 20 and 80 years and/or between the ages of 40 and 50 years. If you are put in different categories for each, whichever is the highest determines your risk category.

Your risk of developing breast cancer between the ages of 20 and 80 years is 16.5%. According to the NICE guidelines this puts you in the **population** risk category.

Your risk of developing breast cancer between ages of 40 and 50 years is 3.1%. According to the NICE guidelines this puts you in the **moderate** risk category.

The table below tells you the ranges for the three risk categories: near population risk, moderate risk and high risk. Near population risk Moderate risk High risk Risk between ages 20 and 80 **Less than 17%** 17% or greater but less than 30% 30% or greater Risk between ages 40 and 50 Less than 3% **3% or greater to 8%** Greater than 8%

	Near population risk	Moderate risk	High risk
Risk between ages 20 and 80	**Less than 17%**	17% or greater but less than 30%	30% or greater
Risk between ages 40 and 50	Less than 3%	**3% or greater to 8%**	Greater than 8%

The graph below shows the different risk categories and your personal risk of developing breast cancer between the ages of 20 and 80 years and 40 and 50 years compared to that of the population.

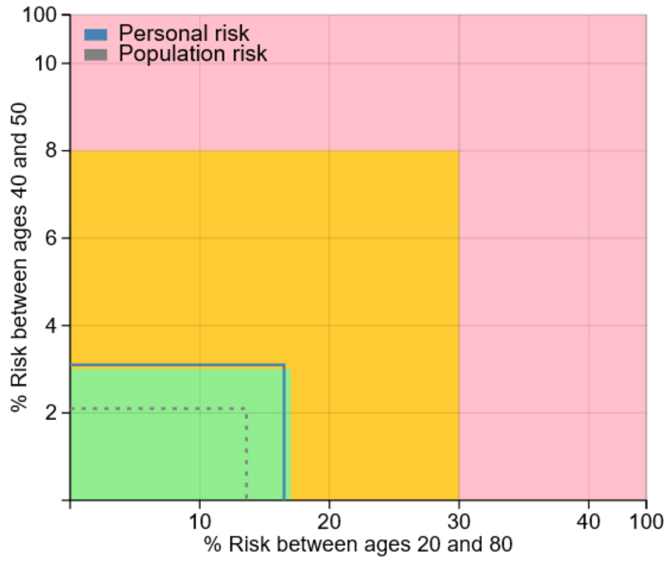

Your risk of developing breast cancer between now and the age of 80 years compared to the population:

Your risk of developing **breast cancer between now and the age of 80 years is 15.4%**, compared to the average population risk of 12.8%. In other words, about 15 out of 100 women with the same risk factors as you will develop breast cancer by the age of 80 years, compared to the population average of about 13 in 100 women in the population.

Your Age (year)	Your Breast Cancer Risk (%)	Population Breast Cancer Risk (%)
41	0.2	0.1
42	0.4	0.3
43	0.6	0.5
44	0.9	0.7
45	1.2	0.9
50	3.1	2.1
55	4.8	3.6
60	6.6	5.2
65	8.7	7
70	11	9
75	13.2	10.9
80	15.4	12.8

Your risk of developing ovarian cancer between now and the age of 80 years compared to the population:

Your risk of developing **ovarian cancer between now and the age of 80 years is 2.3%**, compared to the average population risk of 2.2%. In other words, about 2 out of 100 women with the same risk factors as you will develop ovarian cancer by the age of 80 years, compared to the population average of about 2 in 100 women in the population.

Your Age (year)	Your Ovarian Cancer Risk (%)	Population Ovarian Cancer Risk (%)
41	0	0
42	0	0
43	0.1	0.1
44	0.1	0.1
45	0.1	0.1
50	0.3	0.3
55	0.5	0.5
60	0.8	0.8
65	1.1	1.1
70	1.5	1.4
75	1.8	1.8
80	2.3	2.2

Your risk of carrying a pathogenic gene variant

A pathogenic variant is an alteration in a gene that can contribute to the development of disease. Below you will find an estimate of your likelihood of carrying a pathogenic variant in a gene that has been associated with an increased risk of developing breast and/or ovarian cancer.

Your likelihood of **not** carrying a pathogenic variant in BRCA1, BRCA2, PALB2, CHEK2, ATM, BARD1, RAD51D, RAD51C or BRIP1 is 95.37%

Your estimated likelihood of carrying a pathogenic variant in any of the genes (BRCA1, BRCA2, PALB2, CHEK2, ATM, BARD1, RAD51D, RAD51C or BRIP1) is 4.63%

Your carrier probability for a genetic pathogenic variant in:

- BRCA1 is 1.23%
- BRCA2 is 1.60%
- BRCA1 or BRCA2 is 2.83%
- PALB2 is 0.22%
- CHEK2 is 0.79%
- ATM is 0.38%
- BARD1 is 0.09%
- RAD51D is 0.11%
- RAD51C is 0.07%
- BRIP1 is 0.14%

Note: the genetic pathogenic variant probabilities are calculated by the breast cancer model except for the RAD51D, RAD51C and BRIP1 genes that are calculated by the ovarian cancer model.

SECTION 3: Information more relevant for specialist healthcare professionals

The image below is a visual representation of your family (pedigree) based on the information you provided.

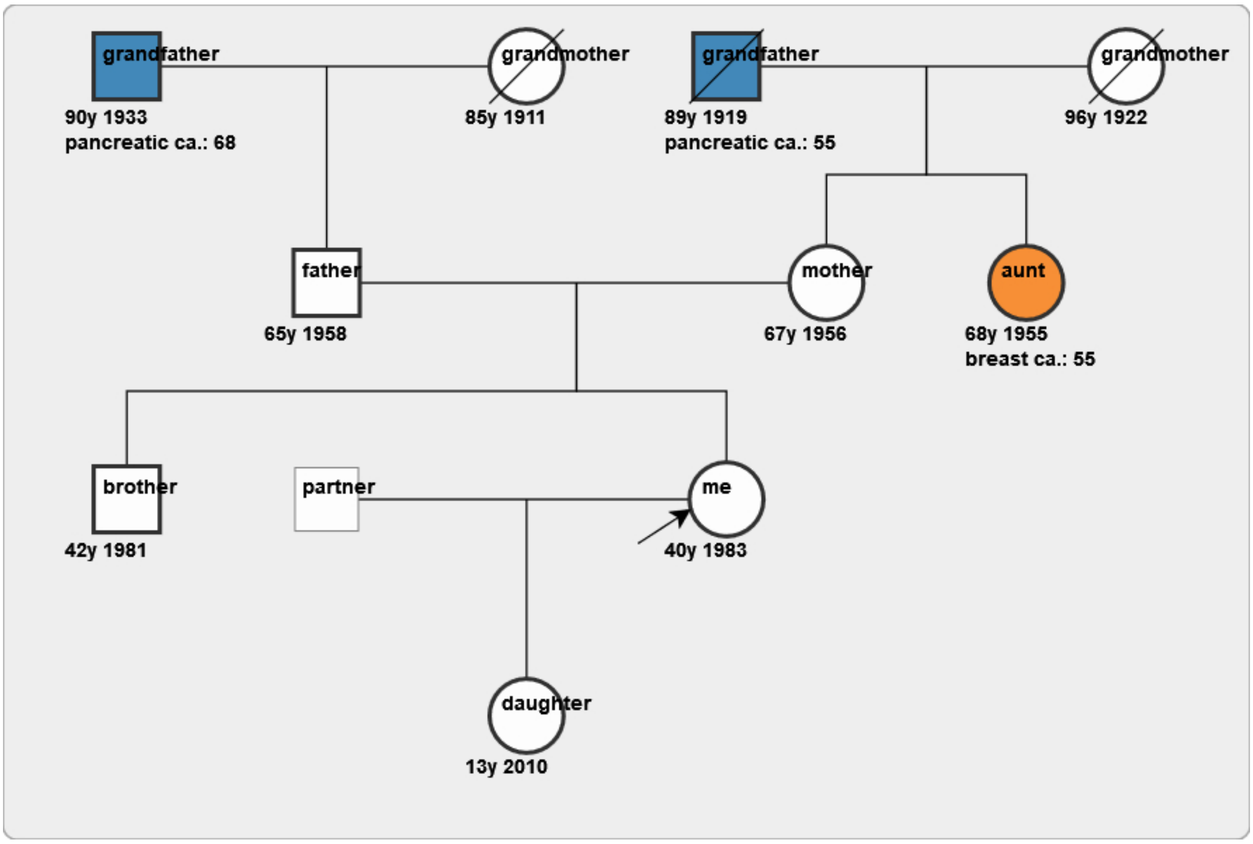

Family pedigree key:

Description	Icon
Male	□
Female	○
Deceased	
Twin	

Cancer	Colour
Breast	
Contralateral breast	
Ovarian	
Prostate	
Pancreatic	

Summary of Genetic Tests and Pathology

No genetic tests or pathology found in the family history.

Breast Cancer Model

The information in the table below was used to calculate your risk of breast cancer. Note that some of these risk factors can increase or decrease risk.

Risk Factor	Value
Age at First Occurrence of Menstruation	-
Number of Children	1
Age of First Live Birth	25-29
Oral Contraception Usage	current
Hormone Replacement Therapy	never/former
Body Mass Index	18.5-<25
Alcohol Intake (grams/day)	15-<25
Age of Menopause	-
Mammographic Density	-
Height (cm)	170

Gene	Mutation frequency	Mutation sensitivity
BRCA1	0.008	0.89
BRCA2	0.006	0.96
PALB2	0.00064	0.92
ATM	0.0018	0.94
CHEK2	0.00373	0.98
BARD1	0.00043	0.89
RAD51C	0.00035	0.78
RAD51D	0.00035	0.86

Note, the following parameter settings were used:

- **Genetic pathogenic variant frequencies:** Ashkenazi
- **Cancer incidence rates:** UK

Please note the model has been developed using data from European ancestry populations.

- **Version:** boadicea model 6.3.1, version 0.6.0; CanRisk v2.4.0-rc1
- **Timestamp:** 2023-09-11T15:48:16.206206+01:00

Breast cancer polygenic scores:

Polygenic scores add together small genetic changes in someone's genetic code (DNA) to help estimate their likelihood of getting a disease, such as breast cancer.
52.8% of people in the population have a **lower** polygenic score than you, and
47.2% of people in the population have a **higher** polygenic score than you.
The graph below might help you visualise this information.

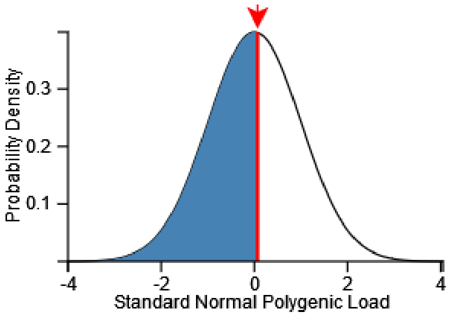

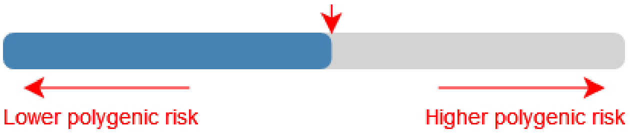

Note: breast cancer polygenic scores are calculated using data from European ancestry populations. The calculated polygenic score z-score is: 0.06939849089331154; the alpha parameter for this polygenic score is: 0.441.

Ovarian Cancer Model

The information in the table below was used to calculate your risk of ovarian cancer. Note that some of these risk factors can increase or decrease risk.

Risk Factor	Value
Number of Children	1
Duration of Oral Contraception Use	10-14
Hormone Replacement Therapy	never
Tubal Ligation	no
Endometriosis	yes
Body Mass Index	22.5-<30
Height (cm)	170

Gene	Mutation frequency	Mutation sensitivity
BRCA1	0.008	0.89
BRCA2	0.006	0.96
RAD51D	0.00035	0.86
RAD51C	0.00035	0.78
BRIP1	0.00071	0.95
PALB2	0.00064	0.92

Note, the following parameter settings were used:

- **Genetic pathogenic variant frequencies:** Ashkenazi
- **Cancer incidence rates:** UK

Please note the model has been developed using data from European ancestry populations.

- **Version:** ovarian model 2.3.1, version 0.6.0; CanRisk v2.4.0-rc1
- **Timestamp:** 2023-09-11T15:48:16.202577+01:00

Summary of cancer disagnoses in your family

The table below summarises information about cancer diagnoses in your family and how old your family members were when they were diagnosed.

Name	Target	IndivID	FathID	MothID	Sex	MZtwin	Dead	Age	Yob	BC1	BC2	OC	PRO	PAN	Ashkn
grandfather	0	SUkD	0	0	M	0	0	90	1933	0	0	0	0	68	0
grandmother	0	cfTD	0	0	F	0	1	85	1911	0	0	0	0	0	0
grandfather	0	HRMw	0	0	M	0	1	89	1919	0	0	0	0	55	0
grandmother	0	XXpO	0	0	F	0	1	96	1922	0	0	0	0	0	0
father	0	m21	SUkD	cfTD	M	0	0	65	1958	0	0	0	0	0	0
mother	0	f21	HRMw	XXpO	F	0	0	67	1956	0	0	0	0	0	0
aunt	0	CiPS	HRMw	XXpO	F	0	0	68	1955	55	0	0	0	0	0
brother	0	Uyaw	m21	f21	M	0	0	42	1981	0	0	0	0	0	0
partner	0	fgNo	0	0	M	0	0	0	0	0	0	0	0	0	0
me	1	ch1	m21	f21	F	0	0	40	1983	0	0	0	0	0	1
daughter	0	pbkk	fgNo	ch1	F	0	0	13	2010	0	0	0	0	0	0

Table key:

- 'Target' is the person undergoing the risk assessment
- 'IndivID' is the unique ID of the family member
- 'FathID' is the unique ID of the father
- 'MothID' is the unique ID of the mother
- 'Sex' is the sex of the person
- 'MZtwin' means identical twins
- 'Dead' is whether the person is dead
- 'Yob' is the person's year of birth
- 'BC1' is the age at first breast cancer diagnosis
- 'BC2' is the age at second (contralateral) breast cancer diagnosis
- 'OC' is age at ovarian cancer diagnosis
- 'PRO' is age at prostate cancer diagnosis
- 'PAN' is age at pancreatic cancer diagnosis
- 'Ashkn' is Ashkenazi status

Advisory Notes

The table below shows where extra information on family members could have increased the accuracy of the model. It shows those family members that have their year of birth and/or age missing. These must be specified in order to be included in a calculation.

Name	Age	Year of Birth
partner	X	X
