# Supplementary material for: Improving the communication of multifactorial cancer risk assessment results for different audiences: a co-design process
Source: J Community Genet. 2024 Sep 25;15(5):499–515. doi: 10.1007/s12687-024-00729-4 (PMC11549070; doi:10.1007/s12687-024-00729-4)
Supplement: Supplementary file 4 — Supplementary file4 (PDF 774 KB) [file 12687_2024_729_MOESM4_ESM.pdf]

CanRisk Report

This report presents the results of your CanRisk assessment and it has three sections.

Section one presents information for you:

- information about your risk of developing breast cancer. These risk results are based on the information you provided about yourself, your family and the results of any genetic tests.

Sections two and three present specific information relevant for healthcare professionals.

Section two presents information for your healthcare professional:

- further information about your risk of developing breast cancer between the ages of 20 and 80 and between the ages of 40 and 50.
- further information about your risk of developing breast cancer between now and the age of 80.
- your risk of carrying a genetic pathogenic variant (or genetic mutation) that is relevant to your risk of developing breast cancer in the future.

Section three presents technical information for specialist healthcare professionals:

- a visual representation of your family (called a pedigree) based on the information you provided.
- a summary of cancer diagnoses in your family based on the information you provided.
- a summary of the other information included in the model used to calculate your breast cancer risks.
- your breast cancer polygenic score.

This report does not include recommendations to manage or lower your risk of developing breast cancer. Your risk is calculated based on the information you have provided and the results of any genetic tests. Note that your risk may change if your risk factors change (more information on risk factors in section 3).

SECTION 1: Information relevant for you

Your Risk of Developing Breast Cancer

Your risk of developing **breast cancer over the next 5 years is 3.2%**. In other words, about 3 out of 100 women with the same risk factors will develop cancer over the next 5 year period. This image might help you visualise this information.

● 3 women are likely to develop breast cancer

○ 97 women are unlikely to develop breast cancer

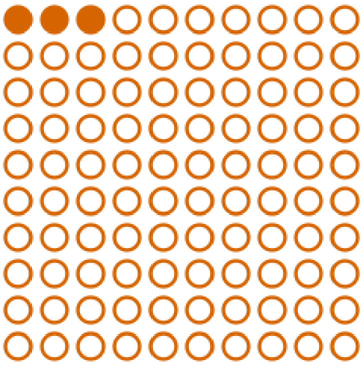

Your risk of developing **breast cancer over the next 10 years is 6.7%**. In other words, about 7 out of 100 women with the same risk factors will develop cancer over the next 10 year period. This image might help you visualise this information.

● 7 women are likely to develop breast cancer

○ 93 women are unlikely to develop breast cancer

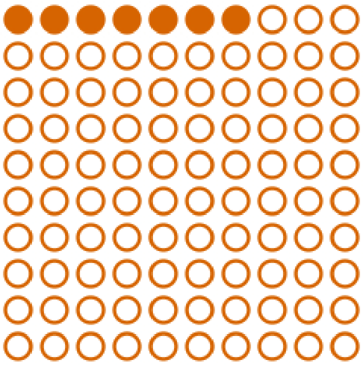

Your risk of developing **breast cancer between now and when you are 80 years old is 23.5%**. In other words, about 24 out of 100 women with the same risk factors will develop cancer by the age of 80. This image might help you visualise this information.

● 24 women are likely to develop breast cancer

○ 76 women are unlikely to develop breast cancer

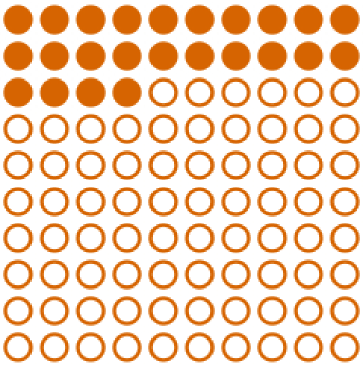

Your Breast Cancer Risks Compared to the Rest of the Population

The graph represents your risk of developing breast cancer between now and the age of 80 years compared to the population. In other words, the graph shows your personal risk of developing breast cancer compared to the average risk of women in the population.

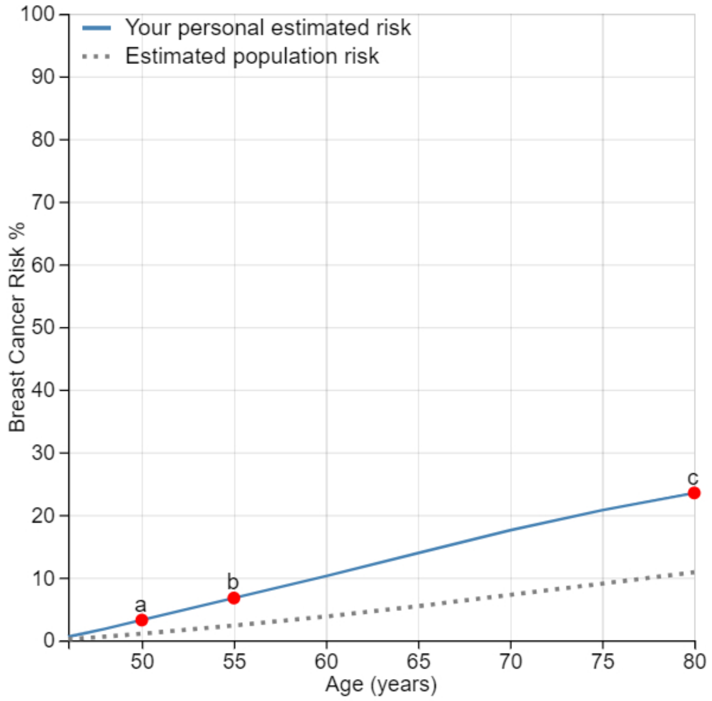

Graph key:

Label	Your estimated breast cancer risk
a	Next 5 year risk is 3.2%
b	Next 10 year risk is 6.7%
c	Risk between now and the age of 80 is 23.5%

Below is an alternative way of visualising your risk of developing breast cancer between now and the age of 80 years compared to the average population risk.

Your risk: 23.5%

● 24 women are likely to develop breast cancer

○ 76 women are unlikely to develop breast cancer

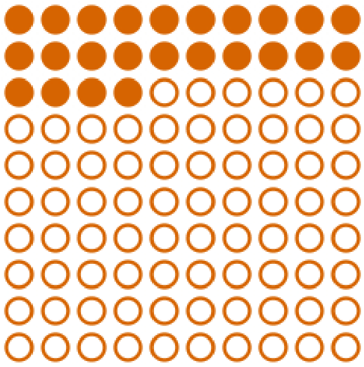

Population risk: 10.9%

● 11 women are likely to develop breast cancer

○ 89 women are unlikely to develop breast cancer

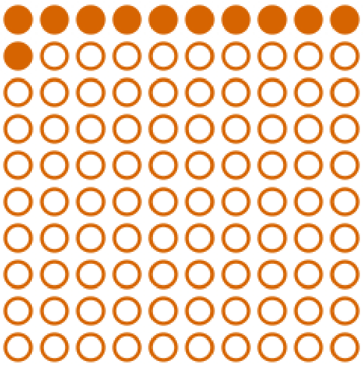

Your Breast Cancer Risk Category (NICE)

Based on your risk assessment and according to the NICE guideline (CG164) you are at **moderate risk of developing breast cancer**. Your risk category may change if your risk factors change.

More information about this is provided in section 2.

Note: NICE is the National Institute for Health and Care Excellence in the UK and it provides evidence-based recommendations.

SECTION 2: Information more relevant for healthcare professionals

Further Information About Your Risk of Developing Breast Cancer

According to the NICE guideline (CG164), your risk category is based on your risk of developing breast cancer between the ages of 20 and 80 years and/or between the ages of 40 and 50 years. If you have different categories for each, whichever is the highest determines your risk category.

- Your risk of developing breast cancer between the ages of 20 and 80 years is 27.3%. According to the NICE guideline (CG164) this corresponds to the **moderate risk** category.
- Your risk of developing breast cancer between ages of 40 and 50 years is 5.5%. According to the NICE guideline (CG164) this corresponds to the **moderate risk** category.

Your risk of developing breast cancer between ages of 40 and 50 years is 5.5%. According to the NICE guideline (CG164) this corresponds to the **moderate risk** category.

The table below presents the ranges for the three risk categories: near population risk, moderate risk and high risk.

	Near population risk (a)	Moderate risk (b)	High risk (c)
Risk between ages 20 and 80	Less than 17%	**17% or greater but less than 30%**	30% or greater
Risk between ages 40 and 50	Less than 3%	**3% or greater to 8%**	Greater than 8%

The graph below shows the different risk categories and your personal estimated risk of developing breast cancer between the ages of 20 and 80 years and 40 and 50 years.

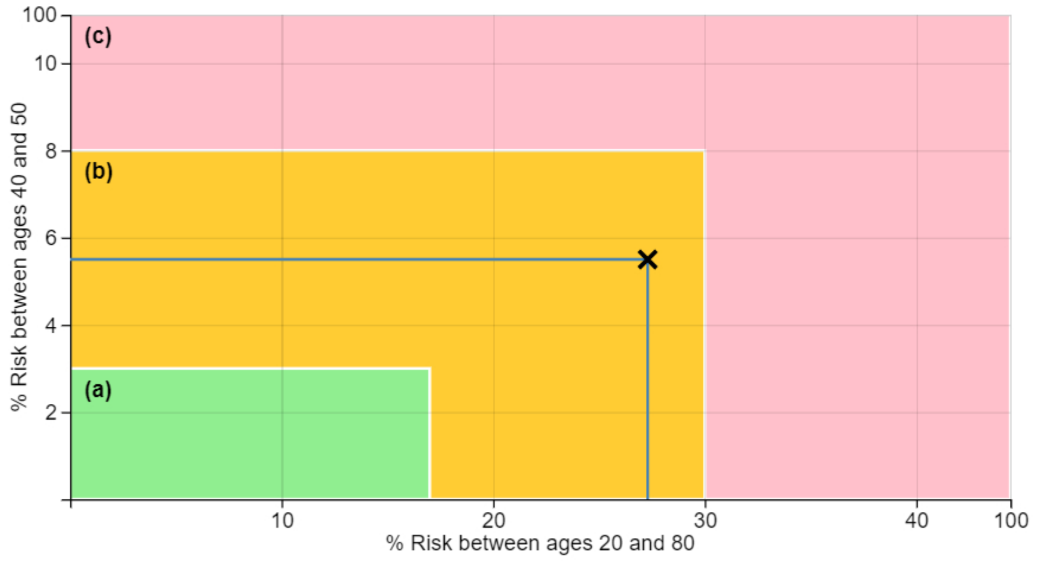

Your risk of developing breast cancer between now and the age of 80 years compared to the population:

Your risk of developing **breast cancer between now and the age of 80 years is 23.5%**, compared to the average population risk of 10.9%. In other words, about 24 out of 100 women with the same risk factors as you will develop breast cancer by the age of 80 years, compared to the population average of about 11 in 100 women in the population.

Your Age (year)	Breast Cancer Risk	Population Breast Cancer Risk (%)
46	0.6	0.2
47	1.2	0.4
48	1.8	0.6
49	2.5	0.8
50	3.2	1.1
55	6.7	2.4
60	10.2	3.8
65	13.9	5.4
70	17.6	7.3
75	20.8	9
80	23.5	10.9

Your Estimated Risk of Carrying an Inherited Pathogenic Gene Variant

A pathogenic variant is an alteration in a gene that can contribute to the development of disease. Below you will find an estimate of your likelihood of carrying a pathogenic variant in a gene that has been associated with an increased risk of developing breast and/or ovarian cancer.

Your likelihood of **not** carrying a genetic pathogenic variant in BRCA1, BRCA2, PALB2, CHEK2, ATM, BARD1, RAD51D, RAD51C or BRIP1 is 97.63%.

Your estimated likelihood of carrying a genetic pathogenic variant in any of the genes (BRCA1, BRCA2, PALB2, CHEK2, ATM, BARD1, RAD51D, RAD51C or BRIP1) is 2.37%.

The table below presents your carrier probability for a genetic pathogenic variant in each of the genes mentioned above.

Gene	Pathogenic Variant Carrier Probability
BRCA1	0.35%
BRCA2	0.16%
BRCA1 or BRCA2	0.51%
PALB2	0.15%
CHEK2	0.71%
ATM	0.37%
BARD1	0.09%
RAD51D	0.15%
RAD51C	0.17%
BRIP1	0.22%

SECTION 3: Information more relevant for specialist healthcare professionals

The image below is a visual representation of your family and how you are related, which is sometimes called a family tree or pedigree. This image is based on the information you provided.

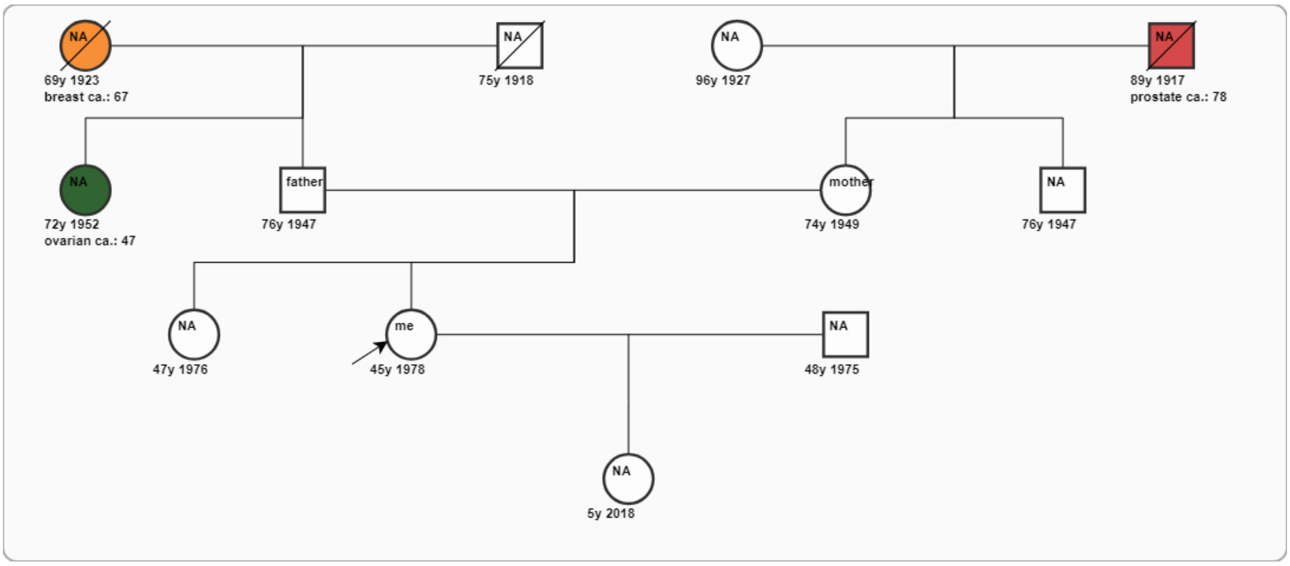

Family pedigree key:

Description	Icon
Male	□
Female	○
Deceased	⌀ ⊠
Twin	

Cancer	Colour
Breast	
Contralateral breast	
Ovarian	
Prostate	
Pancreatic	

Note: the pedigree shows sex assigned at birth

Summary of Genetic Tests and Pathology

No genetic tests or pathology found in the family history.

Breast Cancer Model

The information in the table below was used to calculate your risk of breast cancer. Note that some of these risk factors can increase or decrease risk.

Risk Factor	Value
Age at First Occurrence of Menstruation	14
Number of Children	1
Age of First Live Birth	>29
Oral Contraception Usage	current
Hormone Replacement Therapy	never/former
Body Mass Index	25-<30
Alcohol Intake (grams/day)	>=45
Age of Menopause	40-44
Mammographic Density	-
Height (cm)	168
Polygenic score parameters	z-score=1.6534732826847143; alpha=0.501

Gene	Pathogenic variant frequencies	Test sensitivity
BRCA1	0.0006394	0.89
BRCA2	0.00102	0.96
PALB2	0.00064	0.92
ATM	0.0018	0.94
CHEK2	0.00373	0.98
BARD1	0.00043	0.89
RAD51C	0.00035	0.78
RAD51D	0.00035	0.86

Note, the following parameter settings were used:

- **Genetic pathogenic variant frequencies:** UK
- **Cancer incidence rates:** UK

Please note the model has been developed using data from European ancestry populations.

- **Version:** boadicea model 6.3.2, version 0.6.0; CanRisk v4.0.0
- **Timestamp:** 2024-09-09T10:14:24.576847+01:00

Breast cancer polygenic scores:

Polygenic scores add together small genetic changes in someone's genetic code (DNA) to help estimate their likelihood of getting a disease, such as breast cancer.

95.1% of people in the population have a **lower** polygenic score than you, and

4.9% of people in the population have a **higher** polygenic score than you.

The graph below might help you visualise this information.

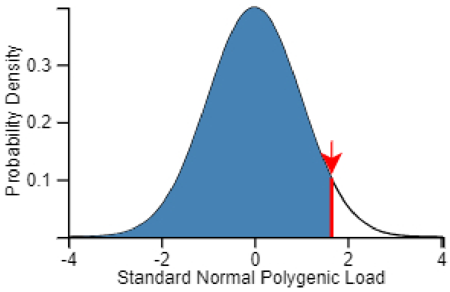

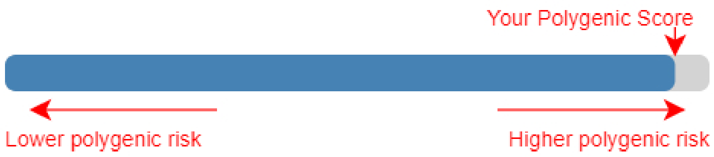

Note: breast cancer polygenic scores are calculated using data from European ancestry populations.

Summary of cancer diagnoses in your family

The table below summarises information about cancer diagnoses in your family and how old your family members were when they were diagnosed.

Name	Target	IndivID	FathID	MothID	Sex	MZtwin	Dead	Age	Yob	BC1	BC2	OC	PRO	PAN	Ashkn
NA	0	KCgm	WeXD	ch1	F	0	0	5	2018	0	0	0	0	0	0
NA	0	Jlbt	m21	f21	F	0	0	47	1976	0	0	0	0	0	0
me	1	ch1	m21	f21	F	0	0	45	1978	0	0	0	0	0	0
NA	0	zOVi	VlEF	AuTE	M	0	0	76	1947	0	0	0	0	0	0
mother	0	f21	VlEF	AuTE	F	0	0	74	1949	0	0	0	0	0	0
NA	0	WeXD	0	0	M	0	0	48	1975	0	0	0	0	0	0
NA	0	gzxS	LPcs	PxSH	F	0	0	72	1952	0	0	47	0	0	0
father	0	m21	LPcs	PxSH	M	0	0	76	1947	0	0	0	0	0	0
NA	0	PxSH	0	0	F	0	1	69	1923	67	0	0	0	0	0
NA	0	LPcs	0	0	M	0	1	75	1918	0	0	0	0	0	0
NA	0	AuTE	0	0	F	0	0	96	1927	0	0	0	0	0	0
NA	0	VlEF	0	0	M	0	1	89	1917	0	0	0	78	0	0

Table key:

- Target - is the person undergoing the risk assessment
- IndivID - is the unique ID of the family member
- FathID - is the unique ID of the father
- MothID - is the unique ID of the mother
- Sex - is the sex of the person
- MZtwin - means identical twins
- Dead - is whether the person is dead
- Yob - is the person's year of birth
- BC1 - is the age at first breast cancer diagnosis
- BC2 - is the age at second (contralateral) breast cancer diagnosis
- OC - is age at ovarian cancer diagnosis
- PRO - is age at prostate cancer diagnosis
- PAN - is age at pancreatic cancer diagnosis
- Ashkn - is Ashkenazi status

Advisory Notes

No advisory notes.
