# Supplementary figures and images for: Improving the communication of multifactorial cancer risk assessment results for different audiences: a co-design process
Source: J Community Genet. 2024 Sep 25;15(5):499–515. doi: 10.1007/s12687-024-00729-4 (PMC11549070; doi:10.1007/s12687-024-00729-4)

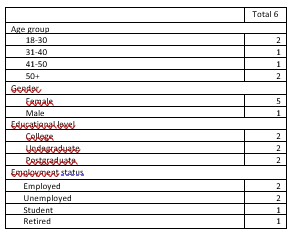

Supplement: Supplementary file 5 — Supplementary file5 (JPG 22 KB) [file 12687_2024_729_MOESM5_ESM.jpg]
